# Characterization of an Nmr Homolog That Modulates GATA Factor-Mediated Nitrogen Metabolite Repression in *Cryptococcus neoformans*

**DOI:** 10.1371/journal.pone.0032585

**Published:** 2012-03-28

**Authors:** I. Russel Lee, Jonathan W. C. Lim, Kate L. Ormerod, Carl A. Morrow, James A. Fraser

**Affiliations:** 1 Australian Infectious Diseases Research Centre, University of Queensland, Brisbane, Queensland, Australia; 2 School of Chemistry and Molecular Biosciences, University of Queensland, Brisbane, Queensland, Australia; University of Minnesota, United States of America

## Abstract

Nitrogen source utilization plays a critical role in fungal development, secondary metabolite production and pathogenesis. In both the *Ascomycota* and *Basidiomycota*, GATA transcription factors globally activate the expression of catabolic enzyme-encoding genes required to degrade complex nitrogenous compounds. However, in the presence of preferred nitrogen sources such as ammonium, GATA factor activity is inhibited in some species through interaction with co-repressor Nmr proteins. This regulatory phenomenon, nitrogen metabolite repression, enables preferential utilization of readily assimilated nitrogen sources. In the basidiomycete pathogen *Cryptococcus neoformans*, the GATA factor Gat1/Are1 has been co-opted into regulating multiple key virulence traits in addition to nitrogen catabolism. Here, we further characterize Gat1/Are1 function and investigate the regulatory role of the predicted Nmr homolog Tar1. While *GAT1/ARE1* expression is induced during nitrogen limitation, *TAR1* transcription is unaffected by nitrogen availability. Deletion of *TAR1* leads to inappropriate derepression of non-preferred nitrogen catabolic pathways in the simultaneous presence of favoured sources. In addition to exhibiting its evolutionary conserved role of inhibiting GATA factor activity under repressing conditions, Tar1 also positively regulates *GAT1/ARE1* transcription under non-repressing conditions. The molecular mechanism by which Tar1 modulates nitrogen metabolite repression, however, remains open to speculation. Interaction between Tar1 and Gat1/Are1 was undetectable in a yeast two-hybrid assay, consistent with Tar1 and Gat1/Are1 each lacking the conserved C-terminus regions present in ascomycete Nmr proteins and GATA factors that are known to interact with each other. Importantly, both Tar1 and Gat1/Are1 are suppressors of *C. neoformans* virulence, reiterating and highlighting the paradigm of nitrogen regulation of pathogenesis.

## Introduction

With over 1,500,000 species estimated to be present in the Earth's biosphere, the kingdom *Fungi* consists of a spectacularly large group of eukaryotes [1]. To successfully thrive in their broad range of niches, many fungal species have evolved mechanisms that enable them to utilize a wide variety of nitrogen sources. The expression of permease and catabolic enzyme-encoding genes needed for the scavenging of most nitrogenous compounds requires activation by global transcription factors belonging to the GATA family. These nitrogen regulatory GATA factors are conserved throughout the phyla *Ascomycota* and *Basidiomycota*. In the model ascomycetes, two positively acting GATA factors *GLN3* and *GAT1* are encoded in the genome of the yeast *Saccharomyces cerevisiae* while single positively acting factors *nit-2* and *areA* are encoded in the genomes of the moulds *Neurospora crassa* and *Aspergillus nidulans*, respectively [2], [3], [4], [5], [6], [7], [8]. Loss-of-function mutations in these ascomycete GATA genes result in an inability to utilize a diverse array of nitrogen sources apart from the readily assimilated and hence generally preferred ammonium or glutamine [9]. Thus, both ammonium and glutamine are metabolites that likely trigger nitrogen metabolite/catabolite repression, resulting in the generation of signals that antagonize activation of secondary (non-preferred) nitrogen gene expression by GATA factors [10], [11], [12].

Although GATA factors are employed to globally control nitrogen metabolism in *S. cerevisiae*, *N. crassa* and *A. nidulans*, there are compelling differences in key aspects of the molecular circuitry that underlies the regulation of these transcription factors in different fungal species. Studies thus far have shown that GATA factor activity is regulated in response to nitrogen availability in the surroundings and/or the nitrogen status of the cell. In *S. cerevisiae*, transcriptional activation of *GAT1* is cross-regulated by both the positively acting factor Gln3 and negatively acting Dal80 [13]. The functions of Gln3 and Gat1 are also regulated posttranscriptionally via protein subcellular localization, through phosphorylation and interaction with the prion-forming glutathione *S*-transferase Ure2 [14], [15], [16], [17].

In *N. crassa*, the function of Nit2 is instead regulated via interaction with a co-repressor protein known as Nmr1 (*N*itrogen *m*etabolic *r*egulation *1*) [18]. Loss-of-function mutations in *nmr-1* result in derepression of a range of secondary nitrogen catabolic genes under normally repressing conditions (nitrogen sufficient conditions, e.g. in the presence of ammonium and/or glutamine) [19]. Unlike *S. cerevisiae* Ure2, there is no evidence suggesting that the structurally unrelated *N. crassa* Nmr1 affects Nit2 subcellular localization. Rather, Nmr1 likely exerts its effect by modulating the *trans*-activation function of Nit2 by interfering with its DNA binding activity [20]. Like *N. crassa* Nit2, the function of *A. nidulans* AreA is also regulated by an Nmr ortholog, NmrA [12], [21], [22], [23]. In addition, AreA activity is further controlled by autogenous regulation and control of transcript stability mediated through an element in the 3′ untranslated region of the *areA* mRNA [23], [24], [25]. These pioneering studies of nitrogen metabolism in *S. cerevisiae*, *N. crassa* and *A. nidulans* have played a crucial role in our current understanding of gene regulation in eukaryotes.

Over the past decade, interest in nitrogen regulation has expanded to encompass a number of important human fungal pathogens, implicating gene regulation by nitrogen availability in virulence. For example, during nitrogen limitation, *Candida albicans* dimorphic transition from the budding yeast to filamentous growth form that facilitates tissue invasion is dependent on the Mep2 ammonium permease, whose expression is regulated by the GATA factors Gln3 and Gat1 [26], [27]. Both *gln3Δ* and *gat1Δ* mutants exhibit reduced virulence in a murine model of disseminated candidiasis [28], [29]. Likewise, the GATA factor AreA enables nutritional versatility that is a key attribute influencing the ability of *Aspergillus fumigatus* to cause disease [30]. Consistent with this notion, the AreA ortholog in *Penicillium marneffei* has recently been proposed to contribute to pathogenicity by regulating the production of extracellular proteases that are potential virulence factors [31]. Impact of nitrogen regulation on virulence is not merely limited to members of the *Ascomycota* but is also observed in the basidiomycete *Cryptococcus neoformans*, a species that causes life-threatening meningoencephalitis predominantly in immunocompromised individuals [32]. For instance, apart from nitrogen catabolism, the GATA factor Gat1/Are1 regulates multiple virulence attributes including infectious basidiospore production, capsule biosynthesis, high temperature growth and melanin pigment formation [33], [34].

Gaining insights into the molecular mechanism governing the regulation of GATA factor activity is therefore important for the understanding of how these pathogenic fungi establish disease in a mammalian host. In *C. albicans*, Gln3 is proposed to be regulated by an Ure2-like mechanism, similar to other closely related yeasts [35], [36]. In contrast, AreA of both *A. fumigatus* and *P. marneffei* are likely to be regulated in an Nmr-like fashion, similar to the related moulds [31], [37]. A recent study by Jiang *et al.* suggests that the yeast *C. neoformans* may follow this previously mould-specific paradigm [38]. The potential Nmr homolog Tar1 (*T*emperature *a*ssociated *r*epressor *1*) was first identified in *C. neoformans* through the overproduction of melanin at 37°C in a clone from a random insertional mutagenesis library [38]. The expression of *TAR1* was induced at high temperature (37°C), and Tar1 was found to negatively regulate *LAC1*-encoded laccase that catalyses the formation of melanin in *C. neoformans* [38]. Like *N. crassa* Nmr1 and *A. nidulans* NmrA, Tar1 harbours a predicted canonical Rossmann fold motif that is also found in other co-repressors such as *S. cerevisiae* Gal80 and mammalian CtBP [21], [38], [39], [40], [41], [42], [43].

As part of an ongoing effort to elucidate the global nitrogen regulatory circuit in the most clinically prevalent form of *C. neoformans* (var. *grubii* strain H99), we have investigated the genetic and physical interactions between Tar1 and the GATA factor Gat1/Are1. In addition, we have examined the role of Tar1 in virulence factor expression and pathogenesis. Overall, our study provides evidence of divergence between different fungal species in the evolution of Nmr-associated proteins, and demonstrates the importance of Tar1 in modulating *C. neoformans* virulence.

## Materials and Methods

### Strains and media

All fungal strains used in this study are listed in Table S1, and were grown in YPD (1% yeast extract, 2% Bacto-peptone, 2% glucose) or YNB (0.45% yeast nitrogen base w/o amino acids and ammonium sulfate, 2% glucose, 10 mM nitrogen source) unless specified otherwise. *C. neoformans* biolistic transformants were selected on YPD medium supplemented with 200 µg/mL G418 (Sigma) or 100 µg/mL nourseothricin (Werner BioAgents). Melanin-inducing media using l-3,4-dihydroxyphenylalanine (l-DOPA), norepinephrine or caffeic acid as the laccase substrate supplemented with 10 mM of the specific nitrogen source were prepared as described previously [32], [38], [44], [45], [46]. Unfiltered 1% pigeon guano medium was also prepared as described previously [34], [47]. *Escherichia coli* Mach-1 cells served as the host strain for transformation and propagation of all plasmids using lysogeny broth supplemented with either 100 µg/mL ampicillin (Sigma) or 50 µg/mL kanamycin (Sigma) [48]. *Caenorhabditis elegans* strain N2 was maintained at 15°C and propagated on its normal laboratory food source *E. coli* OP50 cells [49], [50], [51]. Nematode growth medium (NGM) was prepared as described previously [49].

### Bioinformatic analyses

*C. neoformans* genes were identified using annotation from the H99 genome sequence from the Broad Institute (http://www.broadinstitute.org/annotation/genome/cryptococcus_neoformans/MultiHome.html). Gene annotations from the Broad are designated by their nomenclature *“CNAG#####.#”*. Sequence analyses were performed using BLAST and MacVector 9.5 (MacVector Inc, Cary NC) [52]. Sequence alignments were created using ClustalW v1.4 within MacVector [53]. Sequence traces generated at the Australian Genome Research Facility (Brisbane, Queensland) were analysed using Sequencher 4.7 (Gene Codes Corporation, Ann Arbor MI).

### Construction and complementation of *C. neoformans* mutant strains

All primers and plasmids used in this study are listed in Table S2 and S3, respectively. Gene deletion mutants were created using overlap PCR and biolistic transformation as described previously [54]. Briefly, to construct the *tar1Δ* mutant strain in the H99 background, the 1,067 bp *TAR1* (*CNAG04934.2*) coding sequence was replaced with the neomycin phosphotransferase II-encoding selectable marker *NEO* using a construct created by overlap PCR combining a ∼1 kb fragment upstream the *TAR1* start codon, the *NEO* marker and a ∼1 kb fragment downstream the *TAR1* stop codon. Strain H99 genomic DNA and plasmid pJAF1 were used as PCR templates [55]. The construct was transformed into *C. neoformans* cells via particle bombardment and transformants selected on YPD plates supplemented with G418. A similar approach was adopted to delete *TAR1* in the H99 *gat1/are1::NEO* and wild-type KN99**a** strains using the *NAT* selectable marker from pCH233, with transformants selected on YPD supplemented with nourseothricin. Deletion of *TAR1* was confirmed by diagnostic PCR and Southern blot [56]. To complement the H99 *tar1Δ* mutant, the *TAR1* gene including ∼1 kb promoter and terminator was amplified from genomic DNA using high fidelity PCR, cloned into pCR2.1-TOPO (Invitrogen) to give pIRL25, and sequenced. The *TAR1* fragment of pIRL25 was then subcloned into pCH233, creating the complementation construct pIRL26. pIRL26 was subsequently linearised and biolistically transformed into the *tar1Δ* mutant. Stable transformants were selected on YPD supplemented with nourseothricin and complemented strains containing a single copy of the wild-type *TAR1* gene were identified by Southern blot.

### Quantitative real-time PCR

*C. neoformans* strains were grown in YNB or l-DOPA suppflemented with 10 mM of the specified nitrogen source and shaken at 30 or 37°C for 16 hr. For nitrogen starvation cultures, ammonium-grown cells were gently centrifuged at 1,000 rpm for 3 min, the loose pellets washed with YNB, and cells transferred to fresh YNB medium lacking a nitrogen source for an additional 4 hr. Overnight cultures were harvested, cell pellets frozen and lyophilized, total RNA isolated using TRIzol reagent (Invitrogen) and cDNA generated using the SuperscriptIII First-Strand Synthesis System (Invitrogen). Primers for genes *PUT1 (CNAG02049.2)*, *GAT1/ARE1 (CNAG00193.2)*, *TAR1 (CNAG04934.2)*, *LAC1* (*CNAG03465.2)* and *LAC2 (CNAG03464.2)* were designed to span exon-exon boundaries and tested to verify that they bind specifically to cDNA but not genomic DNA. Quantitative real-time PCR (qRT-PCR) was performed using SYBR Green Supermix (Applied Biosystems) and an Applied Biosystems 7900HT Fast Real Time PCR System with the following cycling conditions: denaturation at 95°C for 10 min, followed by amplification and quantification in 45 cycles at 95°C for 15 sec and 60°C for 1 min, with melting curve profiling at 95°C for 2 min, 60°C for 15 sec and 95°C for 15 sec. Dissociation analysis confirmed the amplification of a single PCR product for each primer pair and an absence of primer dimer formation. Relative gene expression was quantified using SDS software 1.3.1 (Applied Biosystems) based on the 2^−ΔΔCT^ method [57]. The housekeeping actin-encoding gene *ACT1* was used as a control for normalization. One-way analysis of variance was performed using the unpaired, two-tailed *t* test in GraphPad Prism Version 5.0c. *P* values of <0.05 were considered statistically significant.

### Nitrogen utilization, toxic analog sensitivity, melanization, high temperature growth and capsule assays

Starter *C. neoformans* cultures were prepared by growth in YPD at 30°C overnight with shaking, diluted to OD_595 nm_ = 0.05 in water, then further diluted tenfold in series. Each diluted cell suspension was then spotted onto YPD or YNB, l-DOPA, norepinephrine and caffeic acid medium supplemented with the specified nitrogen source or toxic analog. Results were imaged after 2–3 days incubation at 30°C (nitrogen utilization and toxic analog sensitivity assays), or both 30 and 37°C (melanization and high temperature growth assays). For capsule assays, cells were scrapped off the YNB plates, stained with India ink (Becton Dickinson), and visualized under a ZEISS Axioplan 2 epifluorescent/light microscope.

### Yeast two-hybrid assay

Yeast two-hybrid experiments were conducted as described previously [20], [58]. Briefly, full-length *GAT1/ARE1* and *TAR1* ORFs were amplified from H99 cDNA template using high fidelity PCR, and cloned into pCR2.1-TOPO to generate pIRL27 and pIRL21, respectively. The inserted products were sequenced to verify the absence of errors. cDNAs of first-third (nucleotides 1–1,284), second-third (nucleotides 1,285–2,562) and final-third (nucleotides 2,563–3,834) *GAT1/ARE1* fragments, as well as full-length *GAT1/ARE1* (3,834 nucleotides) and *TAR1* (867 nucleotides), were amplified from either pIRL27 or pIRL21 using restriction site-incorporating primers, subcloned into both pGBKT7 and pGADT7 to give pIRL28-37, and again sequenced to ensure correct reading frames obtained. The generated prey and bait constructs with different inserts, as well as the relevant positive (pGBKT7-53, pGADT7-T) and negative (pGBKT7, pGADT7) controls, were subsequently co-transformed into the *S. cerevisiae* reporter strain AH109 using the lithium acetate/heat shock method [59]. Transformants were selected on YNB medium lacking leucine and tryptophan. Interaction was assessed by growth in the absence of adenine and histidine (+5 mM 3-Amino-1,2,4-triazole), and β-galactosidase activity [60].

### *C. elegans* killing assays

Starter cultures of *C. neoformans* strains were prepared by growth in YPD at 30°C overnight with shaking. 10 µL overnight cultures were spread onto both brain-heart infusion (BHI) (Becton Dickinson) and pigeon guano agar plates (35 mm), and incubated at 25°C overnight. ∼50 young adult *C. elegans* worms were then transferred from a lawn of *E. coli* OP50 on NGM to BHI and pigeon guano medium-grown *C. neoformans* [34], [61]. Plates were incubated at 25°C and worms examined for viability at 24 hr intervals using a dissecting microscope, with worms that did not respond to a touch with a platinum wire pick considered dead. Each experimental condition was performed in triplicate. Survival was plotted against time, and *P* values were calculated by plotting a Kaplan-Meier survival curve and performing a log-rank (Mantel-Cox) test using Graphpad Prism Version 5.0c. *P* values of <0.05 were considered statistically significant.

### Murine inhalation model of cryptococcosis

For murine virulence assays, *C. neoformans* were used to infect 6 weeks old female BALB/c mice by nasal inhalation [62]. For every tested strain, ten mice were each inoculated with a 50 µL drop containing 5×10^5^ cells. Mice were weighed before infection and daily thereafter; animals were sacrificed using CO_2_ inhalation once their body weight had decreased to 80% of the pre-infection weight. Survival was plotted against time, and *P* values were calculated by plotting a Kaplan-Meier survival curve and performing a log-rank (Mantel-Cox) test using Graphpad Prism Version 5.0c. *P* values of <0.05 were considered statistically significant.

### Ethics statement

This study was carried out in strict accordance with the recommendations in the Australian Code of Practice for the Care and Use of Animals for Scientific Purposes by the National Health and Medical Research Council. The protocol was approved by the Molecular Biosciences Animal Ethics Committee of The University of Queensland (AEC approval number: SCMB/008/11/UQ/NHMRC). Infection was performed under methoxyflurane anaesthesia, and all efforts were made to minimize suffering through adherence to the Guidelines to Promote the Wellbeing of Animals Used for Scientific Purposes as put forward by the National Health and Medical Research Council.

## Results

### Tar1 is not required for nitrogen source utilization

Although the genome of VNI strain H99 of *C. neoformans* contains multiple open reading frames encoding putative GATA factors, only Gat1/Are1 regulates nitrogen catabolism [34]. An equivalent nitrogen regulatory system that is dependent on a single positively acting GATA factor also occurs in *A. nidulans* and *N. crassa* where AreA and Nit2, respectively, have been extensively characterized [2], [3], [8]. Like the ascomycete moulds, a predicted Nmr homolog Tar1 has been identified in *C. neoformans* but curiously, Jiang *et al.* have suggested that Tar1 plays a negative role in nitrogen metabolism as indicated by the *tar1Δ* mutants faster growth on potassium nitrate as compared to wild-type [38]. This observation is counterintuitive as the inability of *C. neoformans* to utilize nitrate is a classic trait that has long been used as a diagnostic tool to identify this fungus; our recent bioinformatic and phenotypic analyses also supported this lack of nitrate utilization [34], [63], [64].

**Figure 1:**
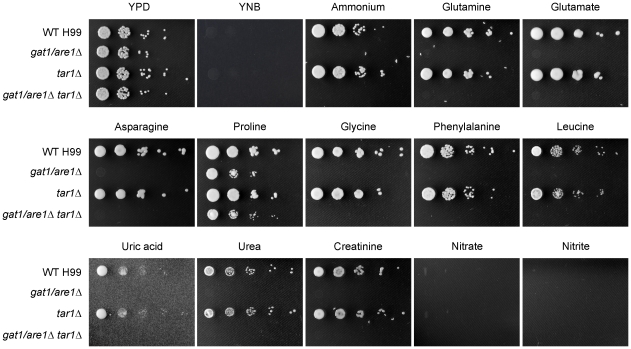
Tar1 is not required for nitrogen source utilization. Tenfold spot dilution assays for nitrogen utilization showed that the *tar1Δ* mutant exhibited wild-type growth on YNB supplemented with ammonium, glutamine, glutamate, asparagine, proline, glycine, phenylalanine, leucine, uric acid, urea and creatinine (10 mM each nitrogen source). In contrast, both the *gat1/are1Δ* and double *gat1/are1Δ tar1Δ* mutants were only able to proliferate on proline as the sole nitrogen source. All tested strains were unable to grow on 7% potassium nitrate or sodium nitrite.

A set of deletion mutants lacking *TAR1*, *GAT1/ARE1* or both was therefore created via homologous recombination in strain H99 to investigate if Tar1 plays a regulatory role in nitrogen utilization (Figure 1). All three generated mutants were viable and had a growth rate indistinguishable from wild-type on rich undefined YPD medium. However, on YNB defined medium supplemented with a panel of different sole nitrogen sources including ammonium, amino acids, purines, nitrate or nitrite, both the *gat1/are1Δ* and double *gat1/are1Δ tar1Δ* mutants only had the ability to proliferate on proline. This result is consistent with previous studies indicating that the *gat1/are1Δ* mutant is unable to utilize ammonium or glutamine but is able to consume proline, a marked difference in phenotype compared to well-characterized ascomycete GATA factor mutants [33], [34]. On the other hand, the *tar1Δ* mutant displayed wild-type growth on all tested nitrogenous compounds except nitrate and nitrite, which all strains were unable to grow on. Together, our results indicate that unlike Gat1/Are1, Tar1 is not required for nitrogen source utilization. Additionally, the similar nitrogen utilization phenotypes displayed by the *gat1/are1Δ* and double *gat1/are1Δ tar1Δ* mutants suggest that if Tar1 does indeed play a regulatory role in these nitrogen catabolic pathways, Gat1/Are1 functions downstream of Tar1.

### Tar1 modulation of nitrogen metabolite repression at the phenotypic level

Gat1/Are1 has been shown to play a role in nitrogen metabolite repression, a regulatory mechanism that enables preferential utilization of readily assimilated nitrogen sources such as ammonium and glutamine [34]. We sought to determine if Tar1 is also functionally associated with this metabolic response. We note that Jiang *et al.* have previously assessed the existence of inappropriate derepression of secondary nitrogen catabolism in the *tar1Δ* mutant by growing the strain solely on potassium chlorate (a toxic analog of potassium nitrate) in the absence of a preferred nitrogen source [38]. However, such sensitivity assays used to determine constitutive expression of non-preferred nitrogen structural genes that are subjected to nitrogen metabolite repression could only be interpreted if a repressing nitrogen source is simultaneously present [21], [23].

**Figure 2:**
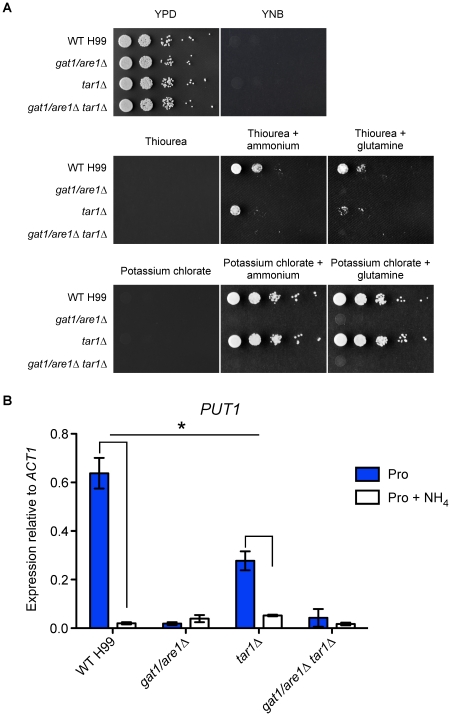
Tar1 plays a role in modulating nitrogen metabolite repression. (A) Tenfold spot dilution assays for nitrogen utilization showed that the *tar1Δ* mutant exhibited slight sensitivity to 5 mM thiourea when compared to wild-type in the simultaneous presence of 10 mM ammonium or glutamine. (B) cDNA from wild-type H99 and the mutant strains grown in YNB supplemented with proline or proline plus ammonium (10 mM each nitrogen source) were amplified via qRT-PCR using primers against the proline oxidase-encoding gene *PUT1* and the control gene *ACT1*. In the wild-type strain, *PUT1* expression was significantly increased in the presence of proline but upregulation was largely abolished when ammonium was also present. In the *tar1Δ* mutant, *PUT1* transcription was intermediate in the presence of proline and upregulation was only partially reduced when ammonium was simultaneously present. Nitrogen metabolite repression sensitivity of *PUT1* in the wild-type strain statistically differs from that in the *tar1Δ* mutant (* denotes *P*<0.05). In the *gat1/are1Δ* and double *gat1/are1Δ tar1Δ* mutants, *PUT1* expression was low under both tested growth conditions. Error bars represent standard errors across three biological replicates.

We therefore tested the *tar1Δ* mutants sensitivity to thiourea (a toxic analog of urea) in the presence of ammonium or glutamine (Figure 2A). Like ascomycete *nmrΔ* mutants that exhibit partially derepressed phenotypes due to derepression of the urea permease, the *tar1Δ* mutant displayed a slight increase in sensitivity relative to wild-type while the negative control *gat1/are1Δ* and double *gat1/are1Δ tar1Δ* mutants were unable to proliferate due to their inability to utilize these nitrogen sources [65]. This result suggests that the *tar1Δ* mutant is metabolizing thiourea to a greater extent than wild-type, despite the simultaneous presence of preferred nitrogen sources. Complementation of the *tar1Δ* mutant subsequently restored the wild-type phenotype (Figure S1). Together, these results indicate that Tar1 plays a negative role in the regulation of genes involved in utilization of more complex nitrogen sources. We also tested sensitivity to potassium chlorate in the presence of ammonium or glutamine and found that the *tar1Δ* mutant grew to the same extent as wild-type, consistent with *C. neoformans* lacking nitrate reductase needed to catabolize potassium chlorate (Figure 2A) [9].

The *S. cerevisiae* Dal80 and Gzf3, and *A. nidulans* AreB proteins are negatively acting GATA factors involved in nitrogen regulation [66], [67]. In *C. neoformans*, six additional GATA factors have been identified: Gat201 and Gat204 work in concert to prevent phagocytosis by macrophages, Bwc2 regulates cell fusion and hyphal development in response to blue light, Cir1 controls iron acquisition, while CNAG04263.2 and CNAG03401.2 have unknown functions [34], [68], [69], [70], [71]. To determine if any of these GATA factors play a negative role in nitrogen catabolism, we tested the *gat201Δ*, *gat204Δ*, *bwc2Δ, cir1Δ*, *CNAG04263.2Δ* and *CNAG03401.2Δ* mutants sensitivity to thiourea in the presence of ammonium or glutamine (not shown). The six GATA deletion mutants exhibited wild-type sensitivity, suggesting that negatively acting nitrogen regulatory GATA factors are likely to be a unique evolutionary feature of the ascomycetes. Consistent with this notion, all seven *C. neoformans* GATA factors including Gat1/Are1, lack putative leucine zippers that are present in the negative acting *S. cerevisiae* Dal80 and Gzf3, or *A. nidulans* AreB. Overall, our analysis thus far revealed that only Tar1 plays a negative role in secondary nitrogen catabolism.

### Tar1 modulation of nitrogen metabolite repression at the transcriptional level

We sought to provide support for our sensitivity assays and prove that Tar1 is implicated for the response to nitrogen metabolite repression in a range of metabolic pathways. We therefore analysed the transcriptional regulation of the proline oxidase-encoding gene *PUT1* using qRT-PCR on RNA extracted from the wild-type, *tar1Δ*, *gat1/are1Δ* and double *gat1/are1Δ tar1Δ* mutant strains grown in YNB supplemented with proline or proline plus ammonium (Figure 2B). The proline catabolic pathway was chosen to be examined as this traditionally non-preferred nitrogen source supports the most robust growth in the *gat1/are1Δ* and double *gat1/are1Δ tar1Δ* mutants, thus enabling the isolation of RNA from a defined medium. Furthermore, *PUT1* has previously been shown to be sensitive to nitrogen metabolite repression, providing an ideal condition to test how Tar1 and Gat1/Are1 are affiliated to this regulatory control mechanism [34].

As expected, high levels of *PUT1* expression were observed when the wild-type strain was cultured in proline as the sole nitrogen source, and this upregulation was largely abolished (∼30-fold difference) with the simultaneous presence of ammonium. In the *tar1Δ* mutant, intermediate levels of *PUT1* mRNA were observed in the presence of proline, and this upregulation was only partially reduced (∼5-fold difference) when ammonium was simultaneously present. While the 2–3-fold more *PUT1* transcript produced by the *tar1Δ* mutant relative to wild-type when grown in proline plus ammonium was anticipated, the 2–3-fold less *PUT1* expression of the *tar1Δ* mutant compared to wild-type in the sole presence of proline was surprising (WT nitrogen metabolite repression sensitivity vs *tar1Δ* nitrogen metabolite repression sensitivity, *P* = 0.0195). The former indicates that *PUT1* transcription in the *tar1Δ* mutant was not completely repressed in the presence of a preferred nitrogen source, and reiterates the fact that Tar1 prevents the expression of secondary nitrogen structural genes under repressing conditions. The latter, intriguingly, suggests that Tar1 also plays a positive role in the activation of secondary nitrogen structural genes under non-repressing conditions, despite the fact that the *tar1Δ* mutant exhibited indistinguishable phenotypic growth rate relative to wild-type on various nitrogen sources (Figure 1). Tar1 therefore contributes to the expression of nitrogen catabolism associated genes, however, it does not overcome the requirement for Gat1/Are1.

### The expression of *GAT1/ARE1*, but not *TAR1*, is regulated in response to nitrogen availability

**Figure 3:**
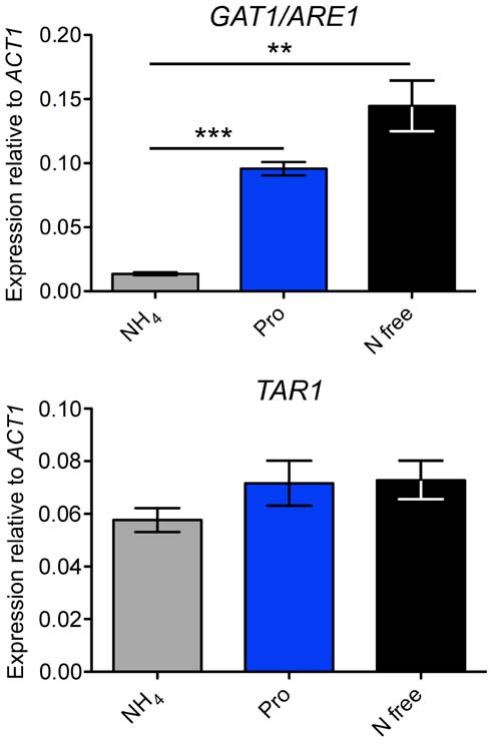
The expression of *GAT1/ARE1*, but not *TAR1*, is regulated in response to nitrogen availability. cDNA from wild-type H99 grown in YNB supplemented with ammonium, proline (10 mM each nitrogen source) or no nitrogen source were amplified via qRT-PCR using primers against *GAT1/ARE1*, *TAR1* and the control gene *ACT1*. In the presence of a preferred nitrogen source (ammonium), the expression of *GAT1/ARE1* was low but in the presence of a traditionally non-preferred nitrogen source (proline), or under nitrogen starvation conditions, *GAT1/ARE1* transcription was significantly upregulated (** denotes *P*<0.01, *** denotes *P*<0.001). In contrast, *TAR1* expression was relatively unaltered under these different nitrogen growth conditions. Error bars represent standard errors across three biological replicates.

In *A. nidulans*, NmrA modulation of AreA activity is finely tuned; this well-coordinated control mechanism begins at the transcriptional level as expression of *areA* and *nmrA* are inversely regulated in response to the quality of the nitrogen source available [37]. To determine if a similar phenomenon occurs in *C. neoformans*, we analysed the transcriptional regulation of both *GAT1/ARE1* and *TAR1* using qRT-PCR on RNA extracted from wild-type H99 grown in YNB supplemented with different nitrogen sources (Figure 3). During growth in ammonium, a low level of *GAT1/ARE1* mRNA was observed, while an elevated level (∼7-fold) of *GAT1/ARE1* transcript was present when grown in proline (Pro vs NH_4_, *P* = 0.0003). A marked increase (>10-fold) in *GAT1/ARE1* transcript level was seen when cells were starved for nitrogen (N free vs NH_4_, *P* = 0.003). In stark contrast to the expression profile of *GAT1/ARE1*, the level of *TAR1* transcript was relatively unaltered when cultured under these different nitrogen conditions. Overall, our qRT-PCR data indicates that *GAT1/ARE1* expression is transcriptionally induced in response to nitrogen limitation, whereas *TAR1* transcription is unaffected by nitrogen availability.

### Tar1 plays a dual function in positive and negative regulation of *GAT1/ARE1* expression according to the nitrogen source present

**Figure 4:**
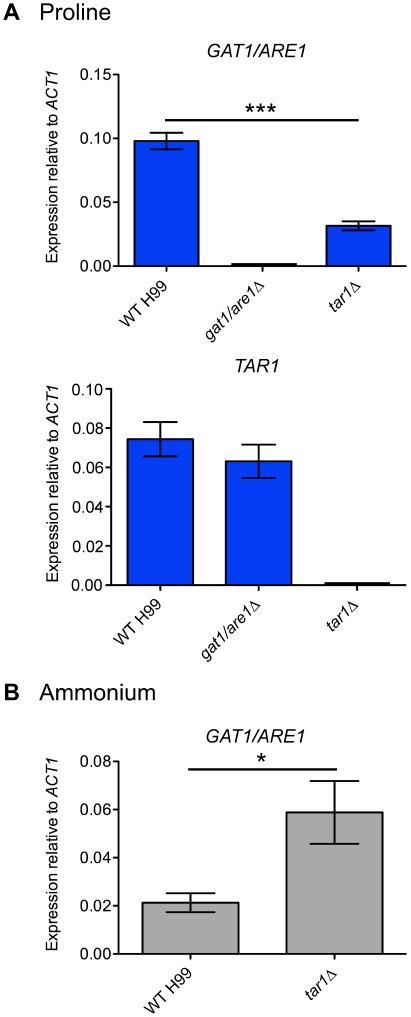
Tar1 plays both positive and negative roles in regulating *GAT1/ARE1* transcription according to the nitrogen source available. (A) cDNA from wild-type H99, *gat1/are1Δ* and *tar1Δ* mutant strains grown in YNB supplemented with 10 mM proline were amplified via qRT-PCR using primers against *GAT1/ARE1*, *TAR1* and the control gene *ACT1*. The expression of *GAT1/ARE1* was significantly lower in the *tar1Δ* mutant compared to wild-type (*** denotes *P*<0.001). Error bars represent standard errors across three biological replicates. (B) cDNA from wild-type H99 and *tar1Δ* mutant strains grown in YNB supplemented with 10 mM ammonium were amplified via qRT-PCR using primers against *GAT1/ARE1* and *ACT1*. The expression of *GAT1/ARE1* was significantly higher in the *tar1Δ* mutant compared to wild-type (* denotes *P*<0.05). Error bars represent standard errors across three biological replicates.

Given that Gat1/Are1 is involved in the utilization of most nitrogen sources, we sought to determine if Tar1 regulates *GAT1/ARE1* expression, and *vice versa*. We first analysed the transcriptional regulation of both *GAT1/ARE1* and *TAR1* using qRT-PCR on RNA extracted from the wild-type, *tar1Δ* and *gat1/are1Δ* mutant strains grown in YNB supplemented with proline (Figure 4A). As expected, no *GAT1/ARE1* or *TAR1* transcripts were detected in the *gat1/are1Δ* and *tar1Δ* mutants, respectively, validating our diagnostic PCR and Southern blot analyses for confirmation of gene deletion. Consistent with nitrogen independence of *TAR1* expression, the *gat1/are1Δ* mutant produced wild-type levels of the *TAR1* transcript. On the other hand, the *tar1Δ* mutant expressed ∼3-fold less *GAT1/ARE1* mRNA compared to wild-type (WT vs *tar1Δ*, *P* = 0.0008). This result provides a plausible explanation for why the *tar1Δ* mutant transcribed less *PUT1* mRNA relative to wild-type in the sole presence of proline (Figure 2B), and indicates that Tar1 positively regulates *GAT1/ARE1* expression under non-repressing conditions.

To provide support for the model that Tar1 plays a dual function in positive and negative regulation of nitrogen catabolism, we analysed the transcriptional regulation of *GAT1/ARE1* using qRT-PCR on RNA extracted from the wild-type and *tar1Δ* mutant strains grown in YNB supplemented with ammonium (Figure 4B). As expected, the wild-type strain produced low levels of *GAT1/ARE1* transcripts under such nitrogen sufficient conditions, while the *tar1Δ* mutant transcribed ∼3-fold more *GAT1/ARE1* mRNA relative to wild-type (WT vs *tar1Δ*, *P* = 0.0481). This result indicates that Tar1 negatively regulates *GAT1/ARE1* expression under repressing conditions. Collectively, Tar1 can function to elicit both activation and repression of *GAT1/ARE1* autoregulation according to the quality of the nitrogen source present.

### Gat1/Are1 lacks the highly conserved extreme C-terminus domain of *A. nidulans* AreA and *N. crassa* Nit2 known to be involved in Nmr recognition

Our phenotypic and transcriptional data suggest that the molecular mechanism of action of Tar1 may operate in a fashion similar to ascomycete Nmr homologs. In *A. nidulans* and *N. crassa*, certain mutations of the GATA DNA-binding domain or extreme C-terminus of AreA and Nit2 lead to partially derepressed phenotypes under nitrogen sufficient conditions similar to that observed for *nmrΔ* mutants, suggesting that these regions are critical for interaction with Nmr proteins and modulation of GATA factor transcriptional activity [18], [23].

**Figure 5:**
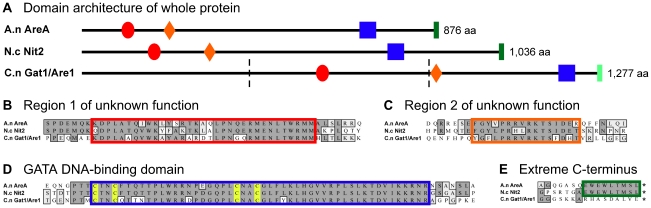
Representative domain architecture and domain sequences of *A. nidulans* AreA, *N. crassa* Nit2 and *C. neoformans* Gat1/Are1. (A) The two conserved regions of unknown function are represented by red ovals and orange diamonds, while the GATA DNA-binding domain is represented by blue squares. The extreme C-terminus domain of AreA and Nit2 (dark green rectangles) is highly conserved but that of Gat1/Are1 has diverged (light green rectangle). Amino acid sequences of all four domains are shown in (B, C, D and E). In addition, the Gat1/Are1 domain architecture is marked with dotted lines to indicate the boundary of the Gat1/Are1 fragments that were tested for protein-protein interaction with Tar1 (see yeast two-hybrid assay section). The universally conserved cysteines involved in zinc ion chelation of the zinc finger are highlighted in yellow.

However, global alignment of *C. neoformans* Gat1/Are1 showed little overall sequence conservation to AreA or Nit2 (15 and 16% identity, respectively) (Figure S2). Importantly, the extreme C-terminus of AreA and Nit2 known to be critical for modulating activities by the co-repressors NmrA and Nmr1, respectively, is absent in Gat1/Are1 (Figure 5A and E) [18], [21], [23]. In fact, the last nine and 12 C-terminus residues of *A. nidulans* AreA are identical to *N. crassa* Nit2 and *Penicillium chrysogenum* GATA factor Nre, respectively (Figure 5E and not shown) [2], [23], [72]. Nonetheless, three notable blocks of conservation do exist between Gat1/Are1 and AreA or Nit2: two regions of unknown function (in AreA and Nit2, these two regions lie near the N-terminus while in Gat1/Are1, they lie further towards the middle or C-terminus region of the protein) (Figure 5A, B and C), and the GATA DNA-binding domain consisting of a zinc finger motif followed by an adjacent basic region (Figure 5A and D). The zinc finger domain has been shown to play a role in the DNA-binding activity of both AreA and Nit2, and the same scenario is likely occurring in *C. neoformans* given the high level of conservation seen among the zinc fingers of Gat1/Are1, AreA and Nit2 [2], [3]. In summary, one of the two regions of ascomycete GATA factors known to be important for interaction with Nmr proteins is absent in Gat1/Are1: the *Cryptococcus* GATA DNA-binding domain is conserved but the extreme C-terminus has diverged.

### Tar1 lacks the conserved C-terminus domain of *A. nidulans* NmrA known to be important for interaction with AreA

We next analysed the sequences of the Nmr homologs of *A. nidulans*, *N. crassa* and *C. neoformans*. In *A. nidulans*, the conserved α-helix at the C-terminus of NmrA is also known to be critical for interaction with AreA [40], [41]. Given that the sequence of *N. crassa* Nmr1 is highly conserved with *A. nidulans* NmrA, it is likely that both these proteins share similar structural features [21], [43]. In contrast, global alignment of *C. neoformans* Tar1 revealed only moderate overall sequence conservation to both NmrA and Nmr1 (19 and 16% identity, respectively) (Figure S3). Tar1 harbours the residues GlyXXGlyXXGly that are predicted to form a Rossmann fold in the N-terminus, while NmrA and Nmr1 contain a slightly diverged canonical Rossmann motif AsnXXGlyXXAla [38], [41], [73]. However, Tar1 lacks the long C-terminus region of NmrA and Nmr1 that is predicted to play a role in interaction with GATA factors. Based on bioinformatics conducted on these GATA factors and Nmr proteins, it was not apparent whether Tar1 is likely to physically interact with and inhibit the function of Gat1/Are1.

### Interaction between Tar1 and Gat1/Are1 was not detectable *in vivo*

The direct interaction of *N. crassa* Nit2 and Nmr1 was originally shown via yeast two-hybrid experiments. The DNA-binding domain or extreme C-terminus of Nit2 fused to the Gal4 activation domain (AD) interacted with an Nmr1-Gal4 DNA-binding domain (BD) fusion protein, with stronger interaction detected when both the DNA-binding domain and C-terminus regions of Nit2 were present [20]. To investigate if a similar situation occurs in *C. neoformans*, we also performed a yeast two-hybrid assay to test if protein-protein interaction occurs between Gat1/Are1 and Tar1 (Table S4). We fused the first-third (nucleotides 1–1,284), second-third (nucleotides 1,285–2,562; contains the first conserved motif of unknown function) and final-third (nucleotides 2,563–3,834; contains the second conserved motif of unknown function, GATA DNA-binding domain and C-terminus) *GAT1/ARE1* fragments, as well as full-length *GAT1/ARE1* and *TAR1* cDNA, to both the *GAL4* AD and BD. Every possible combination of the clones were then expressed in *S. cerevisiae* AH109 in which the *GAL* promoter regulates *ADE2*, *HIS3* and *lacZ* reporter genes. If any protein-protein interaction did exist, it would likely be the final-third Gat1/Are1 fragment and/or full-length Gat1/Are1 (both contains the conserved GATA DNA-binding domain) that may interact with Tar1.

In AH109 strains expressing the first-third fragment or full-length Gat1/Are1-Gal4 BD fusion protein, auto-activation of the bait occurred indicating that an activation domain is present within the nucleotides 1–1,284 of *GAT1/ARE1*. However, in AH109 strains expressing any of the three individual fragments or full-length Gat1/Are1-Gal4 AD fusion protein and Tar1-Gal4 BD fusion protein, cells failed to grow in the absence of adenine and histidine, and did not produce β-galactosidase activity. Our bioinformatics coupled with yeast two-hybrid data therefore suggest that no protein-protein interaction is likely occurring between Gat1/Are1 and Tar1 when expressed in the nucleus of the hemiascomycete *S. cerevisiae*. Additionally, our two-hybrid analysis provides no indication of Gat1/Are1 or Tar1 homodimerization.

### Gat1/Are1 negatively regulates laccase-encoding *LAC1* expression at 37°C

Jiang *et al.* originally identified Tar1 through its negative role in antioxidant melanin production at 37°C on norepinephrine-containing asparagine medium, which eventually led to their finding that Tar1 regulates laccase-encoding *LAC1* expression [38]. The two paralogs *LAC1* and *LAC2* encode laccases that catalyse the formation of melanin in *C. neoformans*, with Lac1 being the main enzyme responsible [45], [74], [75]. Interestingly, our group has also observed that Gat1/Are1 negatively regulates melanin production at 37°C on l-DOPA-containing proline medium [34]. We therefore investigated the genetic epistasis between *TAR1* and *GAT1/ARE1*-regulated production of melanin and expression of laccases.

**Figure 6:**
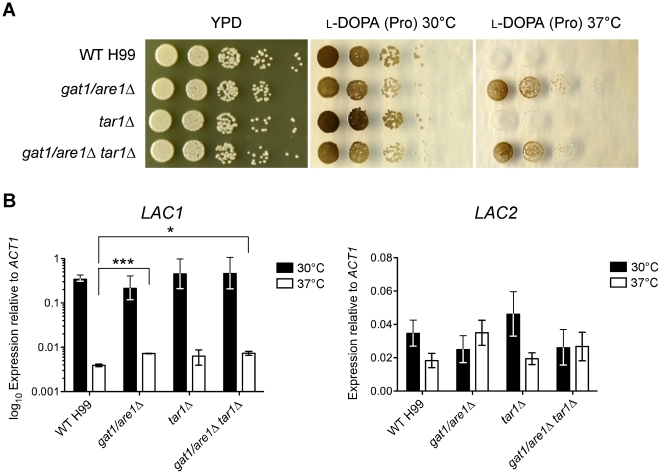
Gat1/Are1 negatively regulates melanin production and *LAC1* expression at 37°C. (A) Tenfold spot dilution assays for melanization showed that the *gat1/are1Δ* and double *gat1/are1Δ tar1Δ* mutants produce more melanin than the wild-type and *tar1Δ* mutant strains on l-DOPA medium supplemented with 10 mM proline at 37°C. (B) cDNA from wild-type H99 and the mutant strains grown in l-DOPA (10 mM proline) at 30 and 37°C were amplified via qRT-PCR using primers against the laccase-encoding genes *LAC1* and *LAC2*, and the control gene *ACT1*. *LAC1* expression was significantly higher in the *gat1/are1Δ* and double *gat1/are1Δ tar1Δ* mutants compared to wild-type or the *tar1Δ* mutant when strains were grown at 37°C (* denotes *P*<0.05, *** denotes *P*<0.001). Error bars represent standard errors across three biological replicates.

Given that the *gat1/are1Δ* and double *gat1/are1Δ tar1Δ* mutants are unable to utilize asparagine as a nitrogen source, we analysed melanin production of the deletion mutants on l-DOPA (proline) agar grown at both 30 and 37°C (Figure 6A). All strains melanized robustly when cultivated at 30°C. However, at 37°C, the *gat1/are1Δ* and double *gat1/are1Δ tar1Δ* mutants produced more melanin than the wild-type and *tar1Δ* mutant strains, reiterating the observation that the *gat1/are1* mutation is dominant over the *tar1* mutation but disagreeing with those seen by Jiang *et al.* [38]. We then analysed the transcriptional regulation of *LAC1* and *LAC2* using qRT-PCR on RNA extracted from the wild-type and mutant strains grown in l-DOPA (proline) at both 30 and 37°C (Figure 6B). As previously reported, transcription of *LAC1* was severely decreased (50–100-fold) at 37°C compared to 30°C [76]. Consistent with our melanin phenotypic plate assays, both the *gat1/are1Δ* and double *gat1/are1Δ tar1Δ* mutants transcribed more (∼2-fold) *LAC1* mRNA as compared to the wild-type or *tar1Δ* mutant strains at 37°C (WT vs *gat1/are1Δ*, *P* = 0.0003; WT vs double *gat1/are1Δ tar1Δ*, *P* = 0.0131). No significant difference in the expression of *LAC1* at 30°C or *LAC2* at 30 and 37°C was observed among the tested strains. Altogether, our data indicates that Gat1/Are1, but not Tar1, represses melanin production and *LAC1* expression at human body temperature when l-DOPA is used as the laccase substrate.

The different substrate used for melanin production may have resulted in conflicting observations made by Jiang *et al.* and our group [38]. We therefore tested melanin production of wild-type H99 and three independent H99 *tar1Δ* mutants, as well as the isogenic wild-type *MAT* **a** strain KN99**a** and six independent KN99**a** *tar1Δ* mutants, at both 30 and 37°C on additional laccase substrates: norepinephrine and caffeic acid, supplemented with asparagine as the nitrogen source (Figure S4). Under all tested growth conditions, no visual difference in melanin production was observed between the wild-type and *tar1Δ* mutant strains. Therefore, in our hands, we are unable to reproduce the increased melanin production and *LAC1* expression phenotypes of the *tar1Δ* mutant as described by Jiang *et al.* [38]. We speculate that the passage of H99 during subculture between laboratories may have possibly caused the phenotypic variation. In support of this notion, Morrow *et al.* have recently reported that various H99 subcultures have differing melanization abilities [77]. Additionally, the indistinguishable growth rate of the *tar1Δ* mutants in comparison to their wild-type H99 or KN99**a** counterparts on YPD medium at 37°C suggests that Tar1 does not play a role in high temperature growth.

Expanding our analysis on virulence factor expression, we examined the polysaccharide capsule that has an antiphagocytic function [78]. We grew the wild-type and *tar1Δ* mutant strains on YNB supplemented with creatinine, uric acid or urea that are known inducers of capsule formation, and found that both the wild-type and *tar1Δ* cells possess equally large capsule on each of these individual medium (not shown) [34]. Given that nitrogen metabolite repression plays a role in the regulation of capsule production, we also examined the capsule of both strains when grown on the same capsule-inducing media but with the simultaneous presence of ammonium (not shown) [34]. Under these growth conditions, the wild-type cells should possess completely repressed (small) capsule since ammonium is a poor inducer of capsule formation, while we were predicting that the *tar1Δ* cells would possess partially repressed (intermediate) capsule due to inappropriate depression of secondary nitrogen (creatinine, uric acid, urea) catabolism [34]. Unexpectedly, the capsule size was equally small and indistinguishable between the wild-type and *tar1Δ* mutant strains under such nitrogen metabolite repression conditions. The subtle role of Tar1 in modulating nitrogen metabolite repression may explain why the *tar1Δ* mutants capsule was not detectably derepressed, as can be seen in the toxic thiourea assays where the *tar1Δ* mutant only exhibited slight sensitivity relative to wild-type (Figure 2A). Taken together, Tar1 does not appear to affect well-established virulence factors including, but not limited to, melanin production, growth at human body temperature and capsule biosynthesis.

### Tar1 is not required for killing of *C. elegans* but modestly represses virulence in a murine inhalation model of cryptococcosis

**Figure 7:**
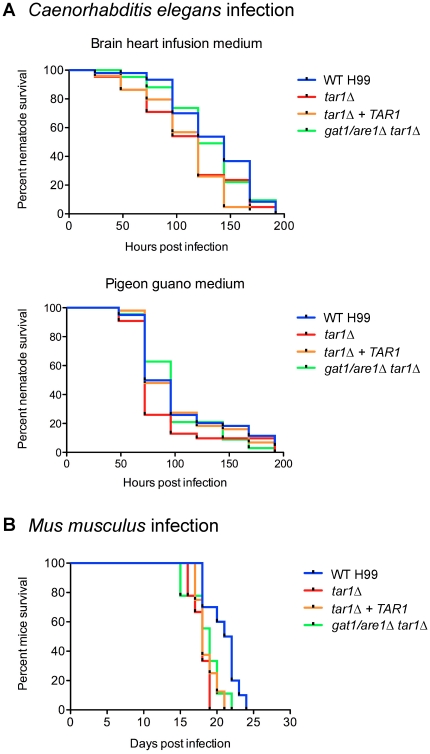
The *tar1Δ* mutant kills *C. elegans* as efficiently as wild-type, but exhibits modestly enhanced virulence in a murine host. (A) *C. elegans* infection: ∼50 nematode worms were transferred to a lawn of wild-type H99, *tar1Δ*, *tar1Δ+TAR1* or double *gat1/are1Δ tar1Δ* cells as the sole food source on both BHI and 1% pigeon guano medium, and survival was monitored at 24 hr intervals. There was no observable difference in *C. elegans* killing by all four strains on both media. (B) *Mus musculus* infection: 10 mice were each intranasally infected with either 5×10^5^ cells of wild-type H99, *tar1Δ*, *tar1Δ+TAR1* or double *gat1/are1Δ tar1Δ* strains, and survival was monitored daily. Mice infected with the *tar1Δ*, *tar1Δ+TAR1* and double *gat1/are1Δ tar1Δ* strains progress to morbidity slightly more rapidly than mice infected with the wild-type strain.

Our overarching interest in *C. neoformans* lies in better understanding its pathogenicity in an animal host. We therefore investigated the role of Tar1 in pathogenesis by performing both *in vitro* and *in vivo* virulence assays. First, we conducted *C. elegans* killing assays using two different media: the standard BHI medium for nematode killing experiments, as well as pigeon guano medium to mimic the *C. neoformans* ecological niche (Figure 7A). Under both growth conditions, killing of *C. elegans* by the *tar1Δ* [LT_50_ (time for half of the worms to die) = 5 and 3 days, for BHI and pigeon guano medium, respectively] and double *gat1/are1Δ tar1Δ* mutants (LT_50_ = 5 and 4 days, for BHI and pigeon guano medium, respectively) was not significantly different to that observed for wild-type (LT_50_ = 6 and 3 days, for BHI and pigeon guano medium, respectively). The undiminished pathogenicity of the double *gat1/are1Δ tar1Δ* mutant is consistent with our previous work showing that Gat1/Are1 does not affect killing of *C. elegans* [34].

Since the *C. elegans* virulence assays were conducted at 25°C, we questioned if Tar1 is required for infection of a host at mammalian body temperature given that the *TAR1* promoter contains putative heat shock sequence elements [38]. To test this hypothesis, we performed a murine inhalation model of cryptococcosis (Figure 7B). Mice infected with the *tar1Δ* mutant succumbed to infection slightly faster (between 16 and 19 days post infection, median survival of 18 days) than mice infected with the wild-type strain (between 18 and 24 days post infection, median survival of 21.5 days) (WT vs *tar1Δ*, *P* = 0.0021). It should be noted that mice infected with the complemented *tar1Δ+TAR1* strain did not progress to morbidity at the same rate as wild-type (between 17 and 21 days post infection, median survival of 18 days). However, the difficulty associated with the restoration of wild-type phenotype of the *tar1Δ* mutant in certain physiological aspect has previously been reported [38]. Alternatively, the role of Tar1 in virulence may be dependent on its expression levels, which could have been adversely affected by integration of *TAR1* into a non-native locus. Mice infected with the double *gat1/are1Δ tar1Δ* mutant also succumbed to infection slightly faster than mice infected with the wild-type strain, consistent with our previous work demonstrating that Gat1/Are1 modestly represses virulence during murine infection (between 15 and 22 days post infection, median survival of 19 days) (WT vs *gat1/are1Δ tar1Δ*, *P* = 0.0342) [34]. Together, these results suggest that both the *tar1Δ* and double *gat1/are1Δ tar1Δ* mutants are slightly more virulent than wild-type during *in vivo* infection. Hence, in addition to fine-tuning regulation of nitrogen catabolism, Tar1 also modulates pathogenesis in a mammalian host.

## Discussion

Nitrogen starvation stimulates the initiation of the infection cycle of the ubiquitous *C. neoformans*, whereby sexual reproduction occurs, leading to the production of potentially infectious basidiospores [69], [79], [80]. This mating process is controlled by multiple signalling cascades including the high affinity ammonium permease Amt2, which is in turn regulated by the GATA factor Gat1/Are1 [34], [81]. In addition to regulating nitrogen catabolism, Gat1/Are1 also impacts multiple virulence attributes such as melanin production, high temperature growth and capsule biosynthesis [33], [34]. Therefore, the underlying mechanism regulating Gat1/Are1 activity in response to nitrogen availability is important for the understanding of the *C. neoformans* life cycle in the environment and successful proliferation during infection of a host.

In the model hemiascomycete *S. cerevisiae*, the function of the GATA factors Gln3 and Gat1 are negatively regulated by interaction with the prion-forming glutathione *S*-transferase Ure2 [14], [15], [16], [17]. As in the system of the filamentous ascomycete *A. nidulans*, there is no genetic or molecular evidence for the existence of an Ure2 homolog in *C. neoformans* [82], [83]. Although we have identified a putative protein sequence (CNAG04110.2) in the H99 genome which contains a glutathione *S*-transferase domain that shows weak overall sequence similarity to Ure2, CNAG04110.2 lacks several features unique to Ure2 including an N-terminus extension and loop region [84], [85].

In *A. nidulans* and *N. crassa*, the function of the GATA factors AreA and Nit2 are negatively regulated by interaction with Nmr proteins that are unrelated to glutathione *S*-transferases [12], [18], [20], [21], [22], [23], [82], [83]. Recently, the group of Jiang *et al.* identified the *C. neoformans* potential Nmr homolog Tar1 during their search for gene disruption mutants that hypermelanize at 37°C [38]. In this study, we further characterized Tar1 function from a different perspective based on our interest in regulation of nitrogen metabolism. Our findings indicate that Tar1 possesses several unique regulatory and functional features in comparison to *A. nidulans* NmrA and *N. crassa* Nmr1.

In *A. nidulans*, *nmrA* expression is low during nitrogen limitation but increases during nitrogen sufficiency [37]. The bZIP transcription factor MeaB activates the expression of *nmrA*, and MeaB homologs are found in all the available filamentous ascomycetes genome databases [37]. In contrast, *TAR1* transcription is unaffected by nitrogen availability and our bioinformatic analyses indicate that the *C. neoformans* H99 genome does not encode an obvious homolog of MeaB. In addition, the promoter region of *TAR1* lacks the conserved element, TTGCACCAT; *in vitro* studies have shown that *A. nidulans* MeaB binds to TTGCACCAT and this binding site is also present in the promoters of NmrA homologs in other filamentous ascomycetes [37]. Instead, Jiang *et al.* have reported that *TAR1* expression is upregulated at high temperature (37°C), consistent with the fact that its promoter contains three TTC/GAA repeats that are putative binding sites for the heat shock factor [38].

However, Tar1 does contain the characteristic NAD/NADH dinucleotide binding motif, GlyXXGlyXXGly, found in members of the short-chain dehydrogenase/reductase family such as the negatively acting Gal80 of *S. cerevisiae*, which acts to block the activation domain of Gal4 required for transcription of galactose-inducible genes [42], [86], [87], [88]. Like the negative transcription regulators *A. nidulans* NmrA and *N. crassa* Nmr1, Tar1 retains the evolutionary conserved function of Nmr proteins of preventing activation of secondary nitrogen catabolism when preferred nitrogenous compounds are present. However, the role of Tar1 in this regulatory aspect is quite subtle, indicative that additional factors are likely operating to modulate nitrogen metabolite repression or to repress Gat1/Are1 activity in *C. neoformans*. For example, in *A. nidulans*, the activity of AreA is also controlled through autogenous regulation and differential transcript stability [23], [24], [25].

Intriguingly, we also discovered a novel role of Tar1 in positively regulating the expression of the proline oxidase-encoding gene *PUT1* under non-repressing conditions. The dual function of Tar1 in fine-tuning nitrogen catabolism appears to be mediated through modulation of *GAT1/ARE1* transcription in response to the quality of the nitrogen source available (Figure S5). Given that Tar1 is not required for nitrogen source utilization, we speculate that only basal-to-intermediate levels of *GAT1/ARE1* transcripts are needed to efficiently activate secondary nitrogen catabolism under non-repressing conditions. The exact mechanism by which Tar1 regulates Gat1/Are1 activity remains unclear as no physical interaction could be detected amongst these two proteins. Since Tar1 does not appear to have a DNA-binding domain, this protein may possibly control the activity of an unknown co-factor(s)/accessory transcription apparatus that in turn influences *GAT1/ARE1* autoregulation in response to nitrogen availability. Further work will be required to dissect the nature and origin of intracellular signals that govern the activity of Gat1/Are1.

Notwithstanding, although a plethora of information about nitrogen metabolism is known in *A. nidulans* and *N. crassa*, studies into the regulation of GATA factor and Nmr protein activity have had their fair share of controversies. For example, while Lamb *et al.* have previously suggested that the N-terminus is required for modulating derepression activity of *A. nidulans* AreA, Caddick and Arst later disagreed with this theory [89], [90]. In more recent times, Lamb *et al.* demonstrated that the extreme nine C-terminus residues of AreA that are highly conserved across a range of filamentous ascomycete homologs does not affect the affinity for NmrA binding, contradicting the findings of Pan *et al.* and Xiao *et al.* [18], [20], [40]. Most recently, Wagner *et al.* asserted that *nmrA* expression in *A. nidulans* is not dependent on MeaB, an observation that contrasts those of Wong *et al.* [37], [91]. Hence, the mechanism of action governing the activity of GATA factors and Nmr proteins in model ascomycetes, too, remain elusive.

Such research into nitrogen regulation is of immense interest as nitrogen source utilization influences key aspects of fungal biology including development, secondary metabolite production and pathogenesis. In the context of virulence, nitrogen regulated pathogenesis has been documented in clinically prevalent human pathogens such as *C. albicans* and *A. fumigatus* [28], [29], [30]. Our analysis of *TAR1* and *GAT1/ARE1* reiterated this dogma; both genes are suppressors of *C. neoformans* virulence. A parsimonious explanation as to why no opposing regulatory effects on pathogenesis were observed between the *tar1Δ* and *gat1/are1Δ* or double *gat1/are1Δ tar1Δ* mutants may relate to the scarce nutrient availability during *in vivo* infection, a condition in which Tar1 is predicted to positively regulate *GAT1/ARE1* activity [34]. Nonetheless, we cannot rule out the possibility that Tar1 is not exclusively involved in regulation of nitrogen catabolism but may also control targets involved in a broader spectrum of metabolic processes. The fact that the *TAR1* promoter contains putative heat shock elements supports this notion [38]. Using whole transcriptome sequencing, we are currently attempting to identify novel virulence and stress adaptation associated genes that are regulated by both Tar1 and Gat1/Are1 during infection of a mammalian host. Indeed, Kronstad *et al.* have recently highlighted transcriptional profiling and genetic studies as invaluable research tools, with the means to provide insights into *C. neoformans* adaptation to key features within a mammalian host environment including nitrogen availability [92].

In summary, there is a pressing need to combat infection from the killer fungus *C. neoformans* that is responsible for >625,000 deaths annually in predominantly AIDS patients [93]. Our study has provided deeper insights into the molecular mechanisms that wire the *Cryptococcus* global nitrogen regulatory circuit. Specifically, we provide an indication of how Tar1 controls Gat1/Are1 activity in response to changes in nitrogen availability, as would occur when *C. neoformans* leaves its purine rich ecological niche of pigeon guano to infect a human host which in comparison is nutrient limiting [94]. Understanding the intricate details of how Gat1/Are1 function is pivotal, as candidate genes in several nitrogen catabolic pathways represent potential drug targets for therapeutic intervention in this important pathogen of humans.

## Supporting Information

Figure S1 **Complementation of toxic analog sensitivity phenotype to wild-type levels upon the re-introduction of** ***TAR1*** **into the** ***tar1Δ*** **mutant.** Tenfold spot dilution assays for nitrogen utilization showed that the *tar1Δ+TAR1* strain exhibited wild-type growth on 5 mM thiourea plus 10 mM ammonium.
(DOC)

Figure S2 **ClustalW multiple sequence alignment of** ***A. nidulans*** **AreA (XP_681936.1),** ***N. crassa*** **Nit2 (P19212.2) and** ***C. neoformans*** **Gat1/Are1 (CNAG00193.2).** Identical amino acid residues are shaded dark grey while similar residues are shaded light grey. Gat1/Are1 shows little overall sequence conservation to AreA and Nit2 at the entire protein level. However, three notable blocks of conversation do exist between Gat1/Are1 and AreA or Nit2: two regions of unknown function (boxed in red and orange) and the GATA DNA-binding domain (boxed in blue). The second conserved motif of unknown function (boxed in orange) was identified through Pustell dot plot protein matrix. The extreme C-terminus domain of AreA and Nit2 (boxed in green) is highly conserved but that of Gat1/Are1 has diverged. Enlarged versions of all four domains are shown in Figure 5B, C, D and E.
(DOC)

Figure S3 **ClustalW multiple sequence alignment of** ***A. nidulans*** **NmrA (AAC39442.1),** ***N. crassa*** **Nmr1 (P23762.2) and** ***C. neoformans*** **Tar1 (CNAG04934.2).** Identical amino acid residues are shaded dark grey while similar residues are shaded light grey. Tar1 shows moderate overall sequence conservation to NmrA and Nmr1. The predicted Rossmann fold motif at the N-terminus is boxed in red. The long C-terminus region of NmrA and Nmr1 is absent in Tar1.
(DOC)

Figure S4 **Multiple independently generated** ***tar1Δ*** **mutants all produced equal amount of pigment melanin in comparison to their wild-type H99 or KN99a counterparts.** The tenfold spot dilution assays for melanization on l-DOPA, norepinephrine and caffeic acid agar (supplemented with 10 mM asparagine as the nitrogen source) were conducted at both 30 and 37°C.
(DOC)

Figure S5 **Scheme representing the dual roles of Tar1 in modulating** ***GAT1/ARE1*** **transcription that in turn influences** ***PUT1*** **expression according to the nitrogen source available.** In the presence of the traditionally non-preferred proline, the Nmr homolog Tar1 positively regulates the transcription of the GATA factor-encoding gene *GAT1/ARE1* that is required for induction of the proline oxidase-encoding gene *PUT1*. In the presence of the preferred ammonium, Tar1 negatively regulates *GAT1/ARE1* transcription leading to reduce levels of *PUT1* expression.
(DOC)

Table S1 **Fungal strains used in this study.**
(DOC)

Table S2 **Primers used in this study.**
(DOC)

Table S3 **Plasmids used in this study.**
(DOC)

Table S4 **Interaction between Tar1 and Gat1/Are1 could not be detected in a yeast two-hybrid assay.** *S. cerevisiae* AH109 was co-transformed with both the bait and prey constructs, and selected for growth on double dropout medium (-Leu -Trp). Interaction (denoted by + symbol) was assessed by growth on quadruple dropout medium (-Leu -Trp -His -Ade), and β-galactosidase activity. − symbol denotes no protein-protein interaction. A.A. denotes auto-activation. N.T. denotes not tested.
(DOC)
