# Supplementary material for: Characterization of an Nmr Homolog That Modulates GATA Factor-Mediated Nitrogen Metabolite Repression in Cryptococcus neoformans
Source: PLoS One. 2012 Mar 28;7(3):e32585. doi: 10.1371/journal.pone.0032585 (PMC3314646; doi:10.1371/journal.pone.0032585)
Supplement: Figure S1 — Complementation of toxic analog sensitivity phenotype to wild-type levels upon the re-introduction of TAR1 into the tar1Δ mutant. Tenfold spot dilution assays for nitrogen utilization showed that the tar1Δ+TAR1 strain exhibited wild-type growth on 5 mM thiourea plus 10 mM ammonium. (DOC) [file pone.0032585.s001.doc]

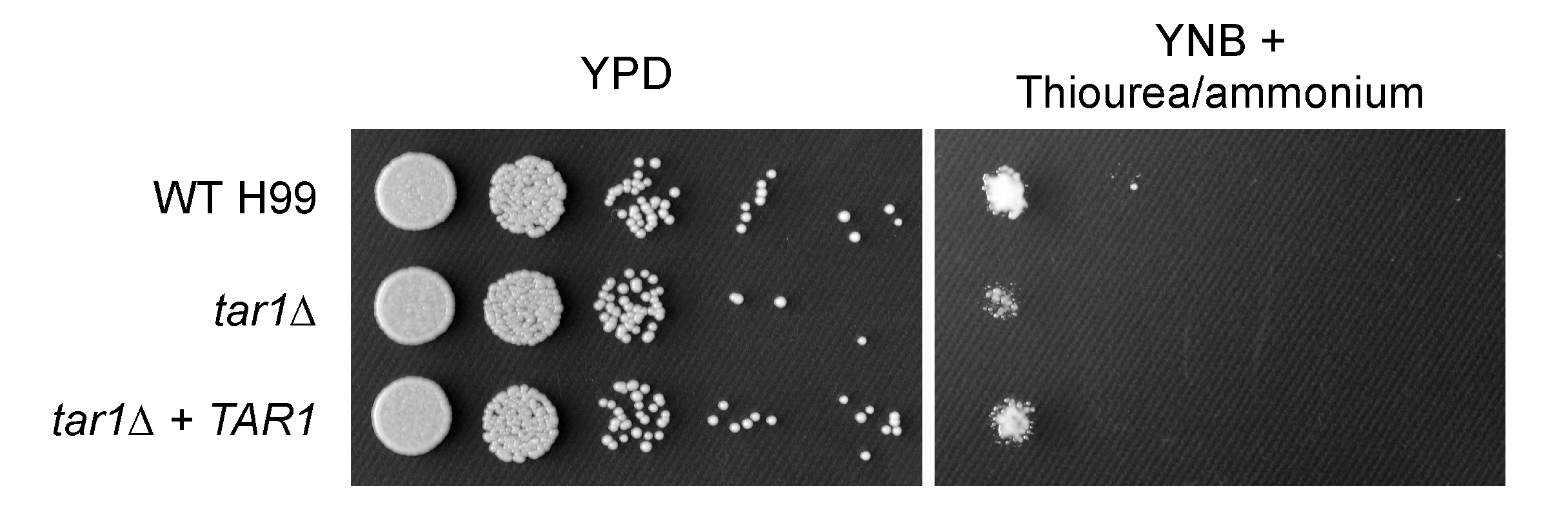


**Figure S1. Complementation of toxic analog sensitivity phenotype to wild-type levels upon the re-introduction of *TAR1* into the *tar1Δ* mutant.** Tenfold spot dilution assays for nitrogen utilization showed that the *tar1Δ + TAR1* strain exhibited wild-type growth on 5 mM thiourea plus 10 mM ammonium.
