# Supplementary material for: Characterization of an Nmr Homolog That Modulates GATA Factor-Mediated Nitrogen Metabolite Repression in Cryptococcus neoformans
Source: PLoS One. 2012 Mar 28;7(3):e32585. doi: 10.1371/journal.pone.0032585 (PMC3314646; doi:10.1371/journal.pone.0032585)
Supplement: Figure S2 — ClustalW multiple sequence alignment of A. nidulans AreA (XP_681936.1), N. crassa Nit2 (P19212.2) and C. neoformans Gat1/Are1 (CNAG00193.2). Identical amino acid residues are shaded dark grey while similar residues are shaded light grey. Gat1/Are1 shows little overall sequence conservation to AreA and Nit2 at the entire protein level. However, three notable blocks of conversation do exist between Gat1/Are1 and AreA or Nit2: two regions of unknown function (boxed in red and orange) and the GATA DNA-binding domain (boxed in blue). The second conserved motif of unknown function (boxed in orange) was identified through Pustell dot plot protein matrix. The extreme C-terminus domain of AreA and Nit2 (boxed in green) is highly conserved but that of Gat1/Are1 has diverged. Enlarged versions of all four domains are shown in Figure 5B, C, D and E. (DOC) [file pone.0032585.s002.doc]

**
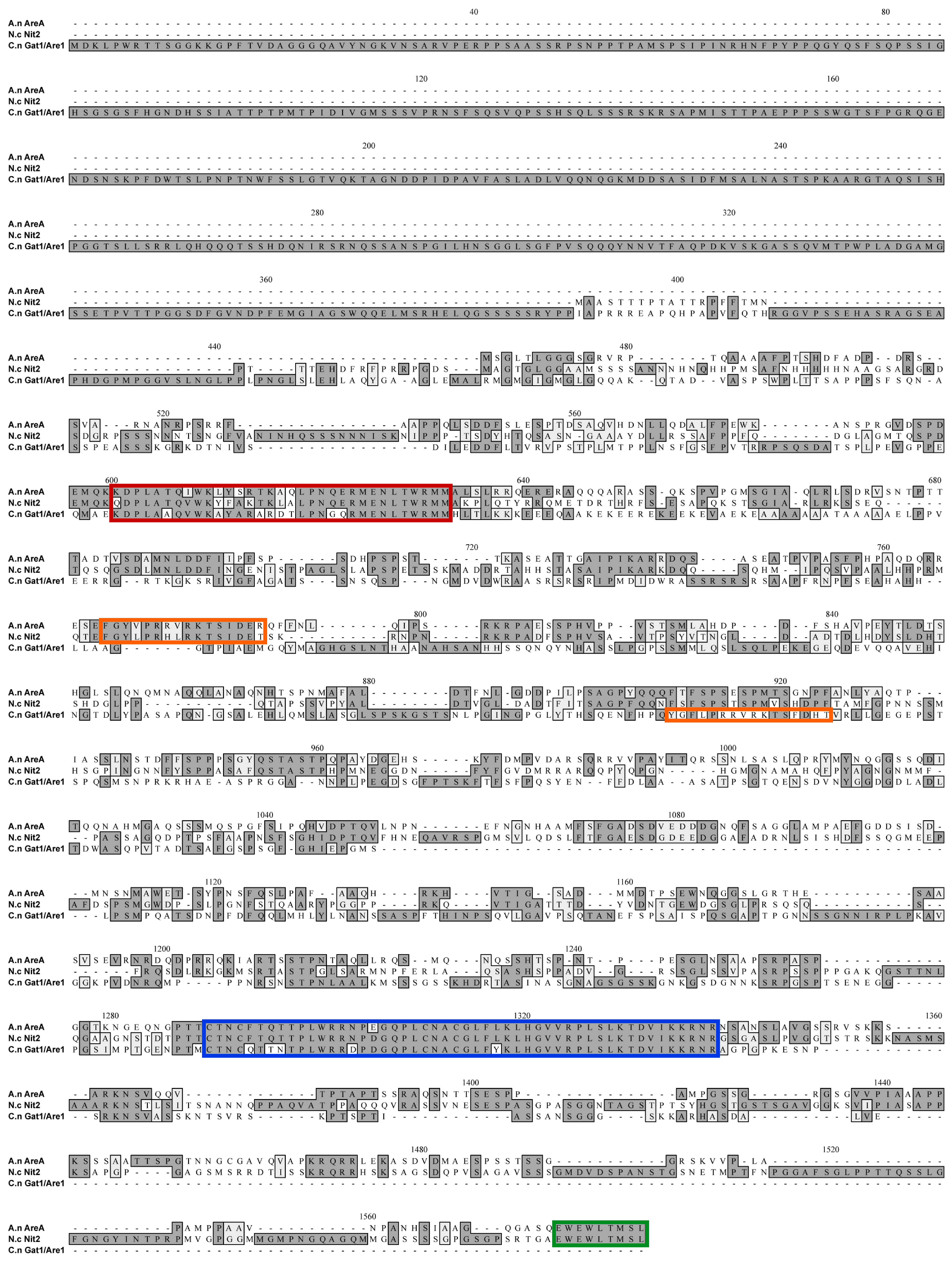
**

**Figure S2. ClustalW multiple sequence alignment of *A. nidulans* AreA (XP_681936.1), *N. crassa* Nit2 (P19212.2) and *C. neoformans* Gat1/Are1 (CNAG00193.2).** Identical amino acid residues are shaded dark grey while similar residues are shaded light grey. Gat1/Are1 shows little overall sequence conservation to AreA and Nit2 at the entire protein level. However, three notable blocks of conversation do exist between Gat1/Are1 and AreA or Nit2: two regions of unknown function (boxed in red and orange) and the GATA DNA-binding domain (boxed in blue). The second conserved motif of unknown function (boxed in orange) was identified through Pustell dot plot protein matrix. The extreme C-terminus domain of AreA and Nit2 (boxed in green) is highly conserved but that of Gat1/Are1 has diverged. Enlarged versions of all four domains are shown in Figure 5B, C, D and E.
