# Supplementary material for: Characterization of an Nmr Homolog That Modulates GATA Factor-Mediated Nitrogen Metabolite Repression in Cryptococcus neoformans
Source: PLoS One. 2012 Mar 28;7(3):e32585. doi: 10.1371/journal.pone.0032585 (PMC3314646; doi:10.1371/journal.pone.0032585)
Supplement: Figure S3 — ClustalW multiple sequence alignment of A. nidulans NmrA (AAC39442.1), N. crassa Nmr1 (P23762.2) and C. neoformans Tar1 (CNAG04934.2). Identical amino acid residues are shaded dark grey while similar residues are shaded light grey. Tar1 shows moderate overall sequence conservation to NmrA and Nmr1. The predicted Rossmann fold motif at the N-terminus is boxed in red. The long C-terminus region of NmrA and Nmr1 is absent in Tar1. (DOC) [file pone.0032585.s003.doc]

**
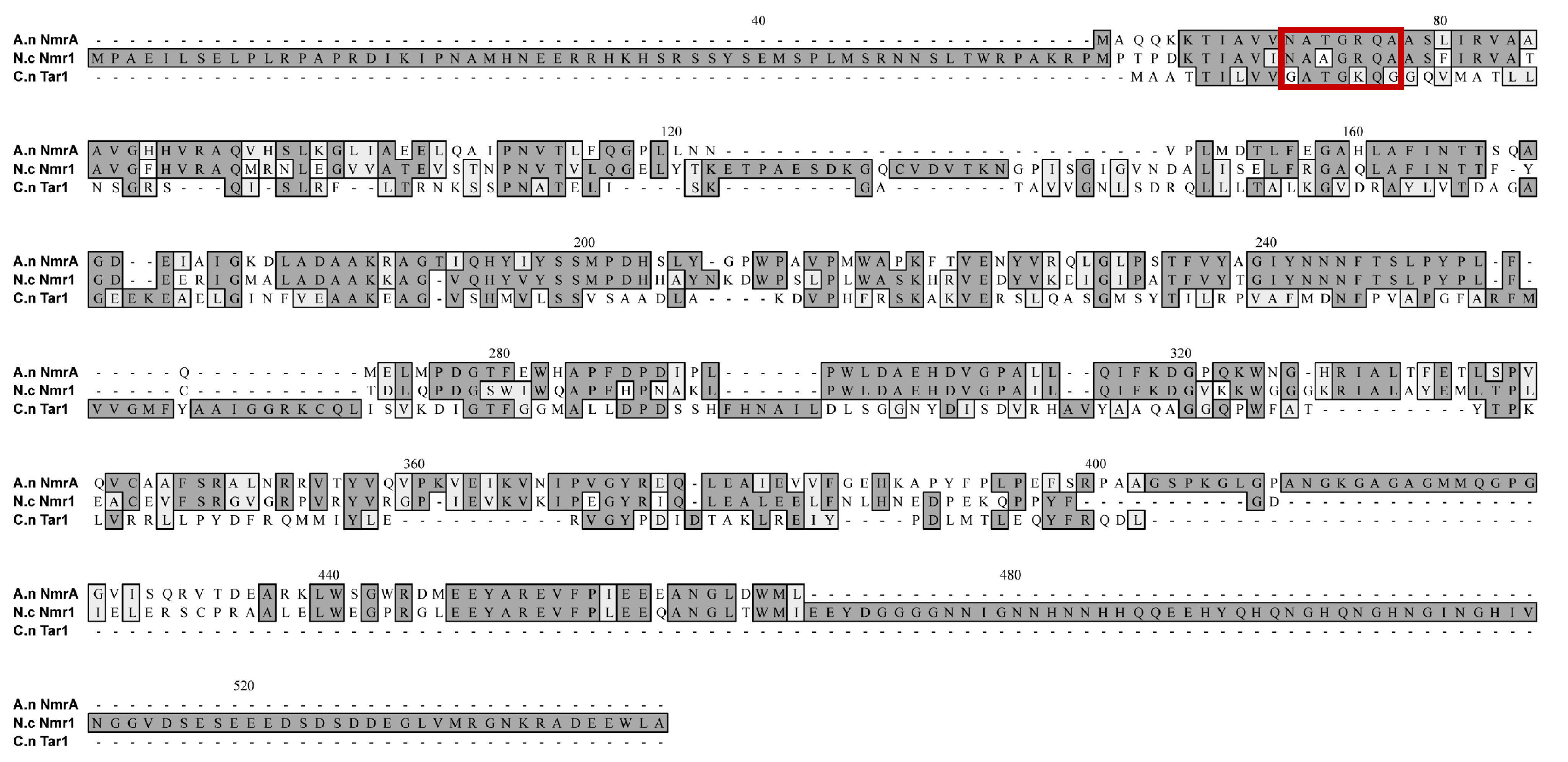
**

**Figure S3. ClustalW multiple sequence alignment of *A. nidulans* NmrA (AAC39442.1), *N. crassa* Nmr1 (P23762.2) and *C. neoformans* Tar1 (CNAG04934.2).** Identical amino acid residues are shaded dark grey while similar residues are shaded light grey. Tar1 shows moderate overall sequence conservation to NmrA and Nmr1. The predicted Rossmann fold motif at the N-terminus is boxed in red. The long C-terminus region of NmrA and Nmr1 is absent in Tar1.
