# Supplementary material for: Characterization of an Nmr Homolog That Modulates GATA Factor-Mediated Nitrogen Metabolite Repression in Cryptococcus neoformans
Source: PLoS One. 2012 Mar 28;7(3):e32585. doi: 10.1371/journal.pone.0032585 (PMC3314646; doi:10.1371/journal.pone.0032585)
Supplement: Figure S4 — Multiple independently generated tar1Δ mutants all produced equal amount of pigment melanin in comparison to their wild-type H99 or KN99a counterparts. The tenfold spot dilution assays for melanization on l-DOPA, norepinephrine and caffeic acid agar (supplemented with 10 mM asparagine as the nitrogen source) were conducted at both 30 and 37°C. (DOC) [file pone.0032585.s004.doc]

**
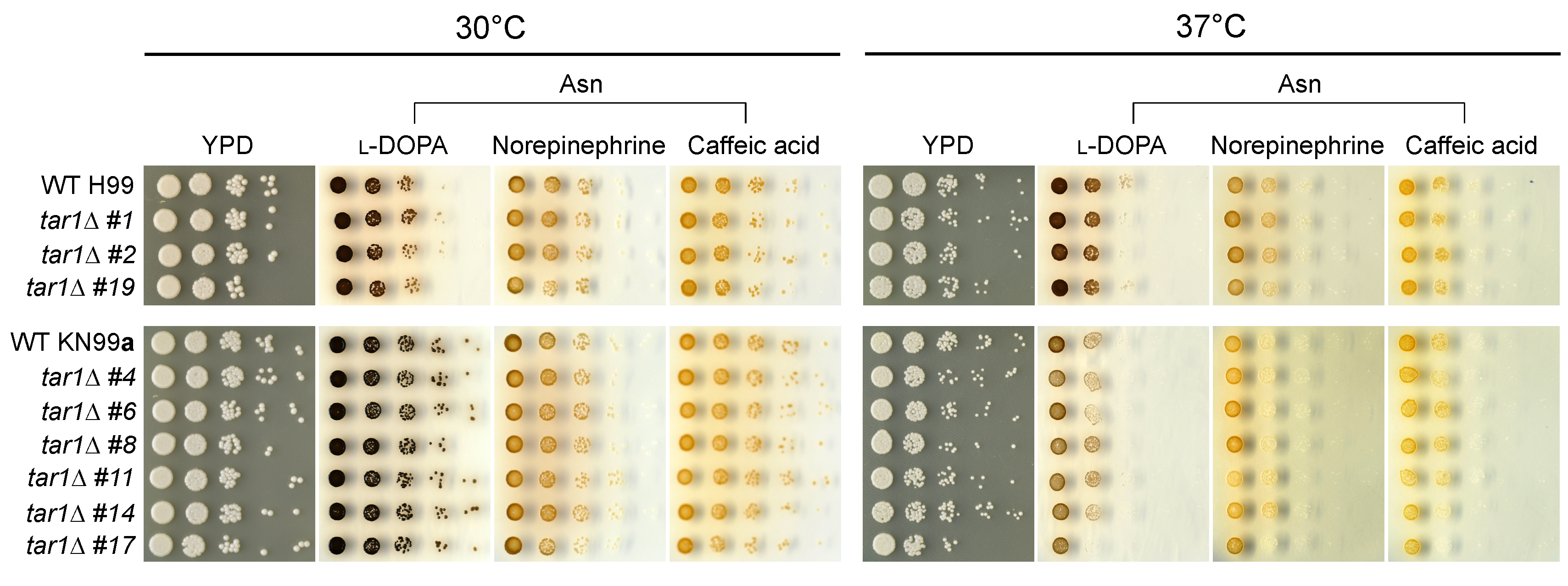
**

**Figure S4. Multiple independently generated *tar1Δ* mutants all produced equal amount of pigment melanin in comparison to their wild-type H99 or KN99a counterparts.** The tenfold spot dilution assays for melanization on L-DOPA, norepinephrine and caffeic acid agar (supplemented with 10 mM asparagine as the nitrogen source) were conducted at both 30 and 37°C.
