# Supplementary material for: Characterization of an Nmr Homolog That Modulates GATA Factor-Mediated Nitrogen Metabolite Repression in Cryptococcus neoformans
Source: PLoS One. 2012 Mar 28;7(3):e32585. doi: 10.1371/journal.pone.0032585 (PMC3314646; doi:10.1371/journal.pone.0032585)
Supplement: Figure S5 — Scheme representing the dual roles of Tar1 in modulating GAT1/ARE1 transcription that in turn influences PUT1 expression according to the nitrogen source available. In the presence of the traditionally non-preferred proline, the Nmr homolog Tar1 positively regulates the transcription of the GATA factor-encoding gene GAT1/ARE1 that is required for induction of the proline oxidase-encoding gene PUT1. In the presence of the preferred ammonium, Tar1 negatively regulates GAT1/ARE1 transcription leading to reduce levels of PUT1 expression. (DOC) [file pone.0032585.s005.doc]

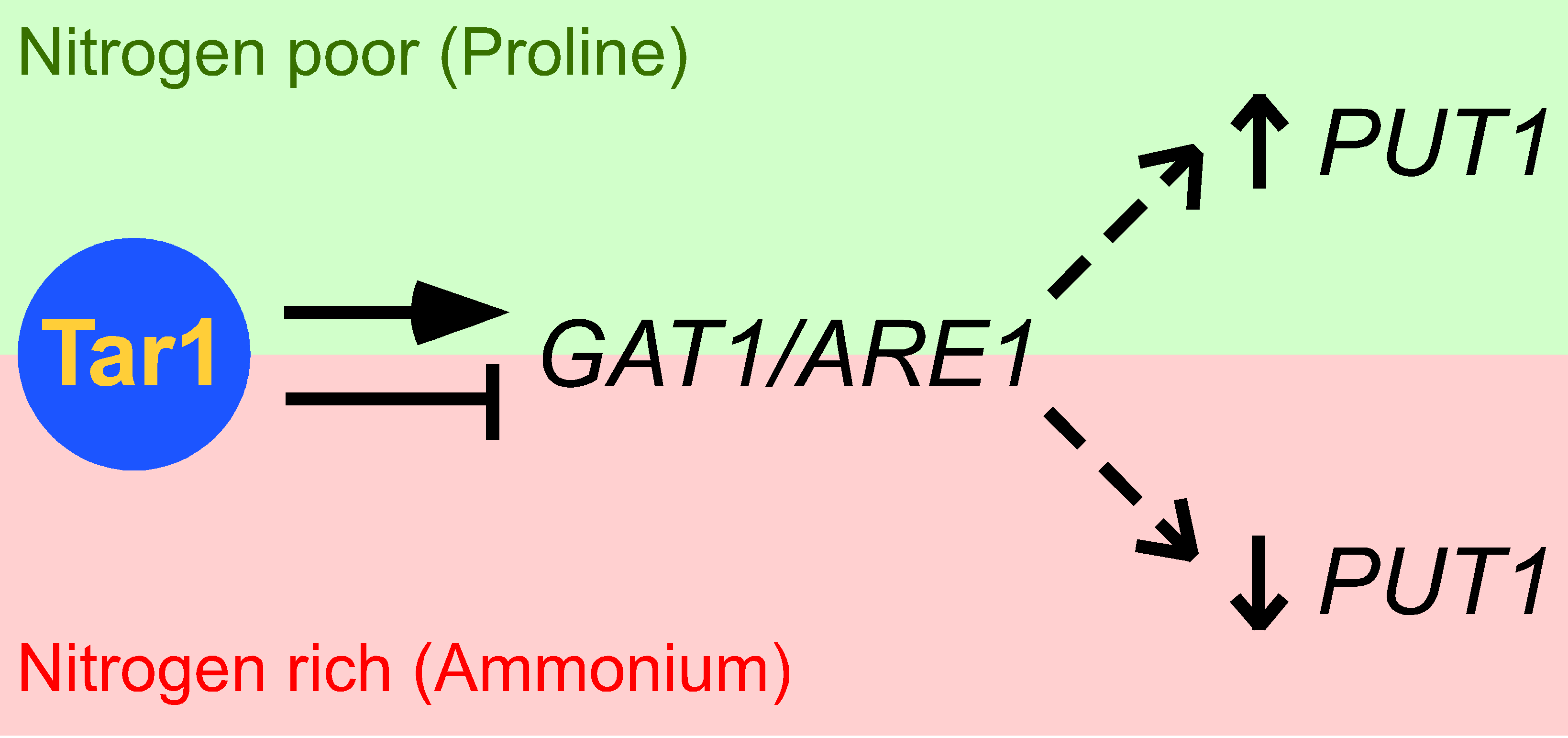


**Figure S5. Scheme representing the dual roles of Tar1 in modulating *GAT1/ARE1* transcription that in turn influences *PUT1* expression according to the nitrogen source available.** In the presence of the traditionally non-preferred proline, the Nmr homolog Tar1 positively regulates the transcription of the GATA factor-encoding gene *GAT1/ARE1* that is required for induction of the proline oxidase-encoding gene *PUT1.* In the presence of the preferred ammonium, Tar1 negatively regulates the transcription of *GAT1/ARE1* leading to reduce levels of *PUT1* expression.
