# Supplementary material for: Characterization of an Nmr Homolog That Modulates GATA Factor-Mediated Nitrogen Metabolite Repression in Cryptococcus neoformans
Source: PLoS One. 2012 Mar 28;7(3):e32585. doi: 10.1371/journal.pone.0032585 (PMC3314646; doi:10.1371/journal.pone.0032585)
Supplement: Table S1 — Fungal strains used in this study. (DOC) [file pone.0032585.s006.doc]

**Table S1.** Fungal strains used in this study.

| **Strain** | **Genotype** | **Original source/reference** |
| --- | --- | --- |
| H99 | *Cryptococcus neoformans* laboratory strain | John Perfect |
| RL1 | *Cryptococcus neoformans* H99 *gat1/are1::NEO* | Lee *et al.* 2011 |
| RL10 | *Cryptococcus neoformans* H99 *tar1::NEO* | This study |
| RL9 | *Cryptococcus neoformans* H99 *gat1/are1::NEO, tar1::NAT* | This study |
| RL14 | *Cryptococcus neoformans* H99 *tar1::NEO + TAR1* | This study |
| RL2 | *Cryptococcus neoformans* H99 *gat201::NEO* | Lee *et al.* 2011 |
| RL3 | *Cryptococcus neoformans* H99 *bwc2::NEO* | Lee *et al.* 2011 |
| RL4 | *Cryptococcus neoformans* H99 *CNAG04263.2::NEO* | Lee *et al.* 2011 |
| RL5 | *Cryptococcus neoformans* H99 *gat204::NEO* | Lee *et al.* 2011 |
| RL6 | *Cryptococcus neoformans* H99 *CNAG03401.2::NEO* | Lee *et al.* 2011 |
| H9CIR4 | *Cryptococcus neoformans* H99 *cir1::NAT* | Won Hee Jung |
| KN99**a** | *Cryptococcus neoformans* congenic strain | Kirsten Nielsen |
| RL11 | *Cryptococcus neoformans* KN99**a** *tar1::NAT* | This study |
| AH109 | *Saccharomyces cerevisiae* matchmaker two-hybrid strain | Clontech |
