# Supplementary material for: Characterization of an Nmr Homolog That Modulates GATA Factor-Mediated Nitrogen Metabolite Repression in Cryptococcus neoformans
Source: PLoS One. 2012 Mar 28;7(3):e32585. doi: 10.1371/journal.pone.0032585 (PMC3314646; doi:10.1371/journal.pone.0032585)
Supplement: Table S2 — Primers used in this study. (DOC) [file pone.0032585.s007.doc]

**Table S2. Primers used in this study.**

| **Primer** | **Purpose** | **Sequence (5’-3’)** |
| --- | --- | --- |
| UQ1070 | *PUT1* qRT-PCR | AAAAGCTAAAGAGAACGACGTTGC |
| UQ1071 | *PUT1* qRT-PCR | TCTCACTCTTTGACTTTGGAGGTTT |
| UQ1634 | *GAT1/ARE1* qRT-PCR | CAATGCGTGTGGACTTTTCTATAAA |
| UQ1635 | *GAT1/ARE1* qRT-PCR | GACCCGCTCGGTTTCTTTTC |
| UQ2067 | *TAR1* qRT-PCR | CAGTGTATCTGCTGCGGACTTG |
| UQ2068 | *TAR1* qRT-PCR | GACATTCCAGAGGCCTGAAGAC |
| UQ1521 | *LAC1* qRT-PCR | TCAATGGACGTGGCCAAAC |
| UQ1522 | *LAC1* qRT-PCR | GCAATTGACTGGCACGTGAA |
| UQ1523 | *LAC2* qRT-PCR | CCTAGTGTATTCGTTCTTTCAAATGACT |
| UQ1524 | *LAC2* qRT-PCR | TGGCCAAAGCCTCAGTAAGG |
| UQ856 | *GAT1/ARE1* deletion / genomic DNA generation | GTTGCGGGATGCTGGTGAAAT |
| UQ857 | *GAT1/ARE1* deletion | AGCTCACATCCTCGCAGCCCTAGCCGCCGCTAGCTG |
| UQ858 | *GAT1/ARE1* deletion | GTGTTAATACAGATAAACCGCGAATGAATGGAAGCA |
| UQ859 | *GAT1/ARE1* deletion / genomic DNA generation | GCCCAGCAACTACCATCGTGT |
| UQ946 | *GAT1/ARE1* deletion | CAGCTAGCGGCGGCTAGGGCTGCGAGGATGTGAGCT |
| UQ947 | *GAT1/ARE1* deletion | TTGCTTCCATTCATTCGCGGTTTATCTGTATTAACA |
| UQ1418 | *TAR1* deletion / genomic DNA generation | TAAAGGTCTGGGCACAGGAAG |
| UQ1419 | *TAR1* deletion | AGCTCACATCCTCGCAGCATTGAATTGAAGAATGTA |
| UQ1420 | *TAR1* deletion | ACTACATTCTTCAATTCAATGCTGCGAGGATGTGAG |
| UQ1421 | *TAR1* deletion | TCTCCCCGGAAGTCTTTTGGTTTATCTGTATTAACA |
| UQ1422 | *TAR1* deletion | TGTTAATACAGATAAACCAAAAGACTTCCGGGGAGA |
| UQ1423 | *TAR1* deletion / genomic DNA generation | CGGCCAGAGAAAGAGTAGCTG |
| UQ18 | Sequencing (M13F pCR2.1-TOPO) | GTAAAACGACGGCCAG |
| UQ19 | Sequencing (M13R pCR2.1-TOPO) | CAGGAAACAGCTATGAC |
| UQ1983 | Sequencing (pGBKT7/pGADT7) | TAATACGACTCACTATAGGGC |
| UQ1984 | Sequencing (pGBKT7) | TTTTCGTTTTAAAACCTAAGAGTC |
| UQ1985 | Sequencing (pGADT7) | AGATGGTGCACGATGCACAG |
| UQ1027 | *GAT1/ARE1* genomic DNA sequencing | GAAAGGCGAATAAAGGCTGTT |
| UQ1028 | *GAT1/ARE1* genomic DNA / cDNA sequencing | TCTGCCGCGTCTTCCCGGCCC |
| UQ1029 | *GAT1/ARE1* genomic DNA / cDNA sequencing | CCAAAAGCTGCTAGGGGCACC |
| UQ1030 | *GAT1/ARE1* genomic DNA / cDNA sequencing | TTGCCAAACGGTTTGTCTCTT |
| UQ1031 | *GAT1/ARE1* genomic DNA / cDNA sequencing | GCAGCCGCAGCAACGGCTGCG |
| UQ1032 | *GAT1/ARE1* genomic DNA sequencing | AAAAAAAAGCATAAATAAAAA |
| UQ1033 | *GAT1/ARE1* genomic DNA / cDNA sequencing | TGACCGCACAGACGTATTTTT |
| UQ1034 | *GAT1/ARE1* genomic DNA / cDNA sequencing | AGCTTTCGGTAATGGCCGGAT |
| UQ1035 | *GAT1/ARE1* genomic DNA / cDNA sequencing | GTGGTCGAAGGATGTCTTCCG |
| UQ1443 | *TAR1* genomic DNA sequencing | CACCAGGCAACACCAGGTTCT |
| UQ1444 | *TAR1* genomic DNA sequencing | CATTCTTCAATTCAATATGGC |
| UQ1445 | *TAR1* genomic DNA sequencing | ATGCTTGTCACAGACCTCTCA |
| UQ1446 | *TAR1* genomic DNA sequencing | GGGGAGAGGGAGGGGGAGACT |
| UQ1735 | *TAR1* genomic DNA sequencing | GCTTGAAAGTGAGTCACTGGC |
| UQ1765 | *TAR1* cDNA sequencing | GACTTCTGCCTTACGACTTCA |
| UQ1766 | *TAR1* cDNA sequencing | CTTGGCGAAGGATGTGCCGCA |
| UQ1716 | *GAT1/ARE1* cDNA generation | CGACAATACGCCTCTCCCCGA |
| UQ1754 | *GAT1/ARE1* cDNA generation | GTCCACTTCTGCCGAAACCAA |
| UQ1755 | *TAR1* cDNA generation | GAAATGTCTTCACATTCGACA |
| UQ1767 | *TAR1* cDNA generation | AGTCTCCCCCTCCCTCTCCCC |
| UQ1971 | Nucleotides 1–1,284 *GAT1/ARE1* cDNA (MfeI site) | GTTAGCCAATTGACTGCGACCAAACAGCAG |
| UQ1973 | Nucleotides 1–1,284 *GAT1/ARE1* cDNA (BglII site) | GGGGCTAGATCTGTCATGAGGAGCTTCAGA |
| UQ1974 | Nucleotides 1,285–2,562 *GAT1/ARE1* cDNA (MfeI site) | GAAGCTCAATTGCCTCATGAAGGCCCCATG |
| UQ1975 | Nucleotides 1,285–2,562 *GAT1/ARE1* cDNA (BglII site) | GAGTTCAGATCTTGGACCATTGATACCAGG |
| UQ1976 | Nucleotides 2,563–3,834 *GAT1/ARE1* cDNA (MfeI site) | GGTCCACAATTGGGCCTCTATACACATAGC |
| UQ1972 | Nucleotides 2,563–3,834 *GAT1/ARE1* cDNA (BglII site) | ATTCGCAGATCTTCATTCAACAAGAGCATC |
| UQ1969 | *TAR1* cDNA (EcoRI site) | TCGACAGAATTCATGGCGGCCACGACCATC |
| UQ1970 | *TAR1* cDNA (BamHI site) | TCTTTTGGATCCCTATAAATCTTGTCGGAA |
