# Supplementary material for: Characterization of an Nmr Homolog That Modulates GATA Factor-Mediated Nitrogen Metabolite Repression in Cryptococcus neoformans
Source: PLoS One. 2012 Mar 28;7(3):e32585. doi: 10.1371/journal.pone.0032585 (PMC3314646; doi:10.1371/journal.pone.0032585)
Supplement: Table S3 — Plasmids used in this study. (DOC) [file pone.0032585.s008.doc]

**Table S3.** Plasmids used in this study.

| **Plasmid** | **Detail** | **Original source** |
| --- | --- | --- |
| pJAF1 | *NEO* selectable marker in pCR2.1-TOPO | James Fraser |
| pCH233 | *NAT* selectable marker in pCR2.1-TOPO | Christina Hull |
| pGBKT7 | Matchmaker two-hybrid *GAL4* BD vector | Clontech |
| pGADT7 | Matchmaker two-hybrid *GAL4* AD vector | Clontech |
| pGBKT7-53 | Murine p53 cDNA in pGBKT7 | Clontech |
| pGADT7-T | SV40 large T antigen cDNA in pGADT7 | Clontech |
| pIRL25 | *TAR1* genomic DNA in pCR2.1-TOPO | This study |
| pIRL26 | *TAR1* genomic DNA in pCH233 | This study |
| pIRL27 | Full-length *GAT1/ARE1* cDNA in pCR2.1-TOPO | This study |
| pIRL21 | Full-length *TAR1* cDNA in pCR2.1-TOPO | This study |
| pIRL30 | Nucleotides 1–1,284 *GAT1/ARE1* cDNA in pGBKT7 | This study |
| pIRL31 | Nucleotides 1,285–2,562 *GAT1/ARE1* cDNA in pGBKT7 | This study |
| pIRL32 | Nucleotides 2,563–3,834 *GAT1/ARE1* cDNA in pGBKT7 | This study |
| pIRL29 | Full-length *GAT1/ARE1* cDNA in pGBKT7 | This study |
| pIRL28 | Full-length *TAR1* cDNA in pGBKT7 | This study |
| pIRL35 | Nucleotides 1–1,284 *GAT1/ARE1* cDNA in pGADT7 | This study |
| pIRL36 | Nucleotides 1,285–2,562 *GAT1/ARE1* cDNA in pGADT7 | This study |
| pIRL37 | Nucleotides 2,563–3,834 *GAT1/ARE1* cDNA in pGADT7 | This study |
| pIRL34 | Full-length *GAT1/ARE1* cDNA in pGADT7 | This study |
| pIRL33 | Full-length *TAR1* cDNA in pGADT7 | This study |
