# Supplementary material for: Characterization of an Nmr Homolog That Modulates GATA Factor-Mediated Nitrogen Metabolite Repression in Cryptococcus neoformans
Source: PLoS One. 2012 Mar 28;7(3):e32585. doi: 10.1371/journal.pone.0032585 (PMC3314646; doi:10.1371/journal.pone.0032585)
Supplement: Table S4 — Interaction between Tar1 and Gat1/Are1 could not be detected in a yeast two-hybrid assay. S. cerevisiae AH109 was co-transformed with both the bait and prey constructs, and selected for growth on double dropout medium (-Leu -Trp). Interaction (denoted by + symbol) was assessed by growth on quadruple dropout medium (-Leu -Trp -His -Ade), and β-galactosidase activity. − symbol denotes no protein-protein interaction. A.A. denotes auto-activation. N.T. denotes not tested. (DOC) [file pone.0032585.s009.doc]

**Table S4.** Interaction between Tar1 and Gat1/Are1 could not be detected in a yeast two-hybrid assay.

| ***GAL4* AD**  ***GAL4* BD** | **Full-length *GAT1/ARE1* (pIRL34)** | **First-third *GAT1/ARE1* (pIRL35)** | **Second-third *GAT1/ARE1* (pIRL36)** | **Final-third *GAT1/ARE1* (pIRL37)** | **Full-length *TAR1* (pIRL33)** | **SV40 large T (+ve control, pGADT7-T)** | **Empty vector (-ve control, pGADT7)** |
| --- | --- | --- | --- | --- | --- | --- | --- |
| **Full-length *GAT1/ARE1* (pIRL29)** | A.A. | A.A. | A.A. | A.A. | A.A. | N.T. | A.A. |
| **First-third *GAT1/ARE1* (pIRL30)** | A.A. | A.A. | A.A. | A.A. | A.A. | N.T. | A.A. |
| **Second-third *GAT1/ARE1* (pIRL31)** | - | - | - | - | - | N.T. | - |
| **Final-third *GAT1/ARE1* (pIRL32)** | - | - | - | - | - | N.T. | - |
| **Full-length *TAR1* (pIRL28)** | - | - | - | - | - | N.T. | - |
| **Murine p53 (+ve control, pGBKT7-53)** | N.T. | N.T. | N.T. | N.T. | N.T. | + | - |
| **Empty vector (-ve control, pGBKT7)** | - | - | - | - | - | - | - |

*S. cerevisiae* AH109 was co-transformed with both the bait and prey constructs, and selected for growth on double dropout medium (-Leu -Trp). Interaction (denoted by + symbol) was assessed by growth on quadruple dropout medium (-Leu -Trp -His -Ade), and β-galactosidase activity. - symbol denotes no protein-protein interaction. A.A. denotes auto-activation. N.T. denotes not tested.
